# Patterns and Predictors of Cognitive Function Among Virally Suppressed Women With HIV

**DOI:** 10.3389/fneur.2021.604984

**Published:** 2021-02-11

**Authors:** Raha M. Dastgheyb, Alison S. Buchholz, Kathryn C. Fitzgerald, Yanxun Xu, Dionna W. Williams, Gayle Springer, Kathryn Anastos, Deborah R. Gustafson, Amanda B. Spence, Adaora A. Adimora, Drenna Waldrop, David E. Vance, Joel Milam, Hector Bolivar, Kathleen M. Weber, Norman J. Haughey, Pauline M. Maki, Leah H. Rubin

**Affiliations:** ^1^Department of Neurology, Johns Hopkins University School of Medicine, Baltimore, MD, United States; ^2^Department of Psychiatry, Johns Hopkins University School of Medicine, Baltimore, MD, United States; ^3^Department of Applied Mathematics and Statistics, Johns Hopkins University, Baltimore, MD, United States; ^4^Division of Biostatistics and Bioinformatics, The Sidney Kimmel Comprehensive Cancer Center, Johns Hopkins University School of Medicine, Baltimore, MD, United States; ^5^Department of Molecular and Comparative Pathobiology, Johns Hopkins University School of Medicine, Baltimore, MD, United States; ^6^Division of Clinical Pharmacology, Johns Hopkins University School of Medicine, Baltimore, MD, United States; ^7^Department of Epidemiology, Johns Hopkins Bloomberg School of Public Health, Baltimore, MD, United States; ^8^Montefiore Medical Center, Albert Einstein College of Medicine, Bronx, NY, United States; ^9^Department of Neurology, State University of New York Downstate Health Sciences University, Brooklyn, NY, United States; ^10^Division of Infectious Disease and Travel Medicine, Department of Medicine, Georgetown University, Washington, DC, United States; ^11^Division of Infectious Diseases, Department of Medicine, University of North Carolina at Chapel Hill, Chapel Hill, NC, United States; ^12^Nell Hodgson Woodruff School of Nursing, Emory University, Atlanta, GA, United States; ^13^School of Nursing, University of Alabama at Birmingham, Birmingham, AL, United States; ^14^Institute for Health Promotion & Disease Prevention Research, University of Southern California, Los Angeles, CA, United States; ^15^Department of Psychiatry & Behavioral Science, University of Miami Miller School of Medicine, Miami, FL, United States; ^16^CORE Center, Cook County Health, Hektoen Institute of Medicine, Chicago, IL, United States; ^17^Department of Psychiatry and Psychology, University of Illinois at Chicago, Chicago, IL, United States

**Keywords:** HIV, cognition, women, heterogeneity, phenotypes, random forest, machine learning

## Abstract

Cognitive impairment remains frequent and heterogeneous in presentation and severity among virally suppressed (VS) women with HIV (WWH). We identified cognitive profiles among 929 VS-WWH and 717 HIV-uninfected women from 11 Women's Interagency HIV Study sites at their first neuropsychological (NP) test battery completion comprised of: Hopkins Verbal Learning Test-Revised, Trail Making, Symbol Digit Modalities, Grooved Pegboard, Stroop, Letter/Animal Fluency, and Letter-Number Sequencing. Using 17 NP performance metrics (T-scores), we used Kohonen self-organizing maps to identify patterns of high-dimensional data by mapping participants to similar nodes based on T-scores and clustering those nodes. Among VS-WWH, nine clusters were identified (entropy = 0.990) with four having average T-scores ≥45 for all metrics and thus combined into an “unimpaired” profile (*n* = 311). Impaired profiles consisted of weaknesses in: (1) sequencing (*Profile-1*; *n* = 129), (2) speed (*Profile-2*; *n* = 144), (3) learning + recognition (*Profile-3*; *n* = 137), (4) learning + memory (*Profile-4*; *n* = 86), and (5) learning + processing speed + attention + executive function (*Profile-5*; *n* = 122). Sociodemographic, behavioral, and clinical variables differentiated profile membership using Random Forest models. The top 10 variables distinguishing the combined impaired vs. unimpaired profiles were: clinic site, age, education, race, illicit substance use, current and nadir CD4 count, duration of effective antiretrovirals, and protease inhibitor use. Additional variables differentiating each impaired from unimpaired profile included: depression, stress-symptoms, income (*Profile-1*); depression, employment (*Profile 2*); depression, integrase inhibitor (INSTI) use (*Profile-3*); employment, INSTI use, income, atazanavir use, non-ART medications with anticholinergic properties (*Profile-4*); and marijuana use (*Profile-5*). Findings highlight consideration of NP profile heterogeneity and potential modifiable factors contributing to impaired profiles.

## Introduction

Early in the HIV epidemic, people with HIV (PWH) frequently exhibited distinct clinical features including cognitive, behavioral, and motor dysfunction characteristic of a subcortical dementia (1, 2). The clinical syndrome was progressive, severe and included slow mental processing, memory impairment, gait disturbance, tremors, apathy, and depressive symptoms. Since the advent of effective and accessible antiretroviral therapy (ART), PWH are living longer and may be more likely to develop comorbidities that include hypertension, diabetes, cardiovascular disease, chronic liver and renal disease, and malignancies (3, 4). Although it remains unclear as to whether these comorbidities accelerate and/or potentiate CNS dysfunction, different combinations of comorbidities are likely to result in diverse patterns of cognitive function. Thus, in PWH there is a need to understand cognitive profiles and their correlates, including sociodemographic, clinical, and behavioral factors in the context of viral suppression. Cognitive phenotyping in NeuroHIV research may facilitate a better understanding of the underlying pathophysiological mechanisms of each specific cognitive profile.

Several studies using different methodological approaches focus on patterns and predictors of cognitive function in PWH (5–7). Cognitive patterns in PWH were first investigated by Lojek and Bornstein (5), who identified four patterns in 162 predominately White (93%), young (mean age = 34 years), and educated (mean years of education = 14) men at various stages of HIV infection. Using dimension reduction (factor analysis) of seven neuropsychological (NP) outcome metrics from 16 tests followed by k-means clustering, the four profiles consisted of (1) a generally unimpaired group; and weaknesses or impairments in (2) only psychomotor speed, (3) only memory and learning, and (4) most domains. A recent cross-sectional study identified three profiles using five cognitive domain T-scores in a latent profile analysis in almost 3,000 predominately White (69%), educated (mean years of education = 15) men with HIV (MWH; 53%) and without HIV from the Multicenter AIDS Cohort Study (MACS; mean age = 40 years) (7). The three profiles included an unimpaired profile, a profile below average on learning and memory, and a profile below average on all domains. Similarly, three profiles were identified using 10 NP outcome metrics in a latent profile analysis in 361 PWH who were predominately men (88%), actively receiving ART (94%) at the Southern Alberta Clinic (6). Again, an unimpaired profile was identified along with a profile with specific weaknesses in executive function and memory and one with more global NP impairment. Notably, each of these studies focused on all or predominately White, educated MWH and included mixed samples of virological suppressed (VS) and non-suppressed (NVS) individuals. Findings in MWH cannot necessarily be generalized to women with HIV (WWH). WWH may be at greater risk for cognitive impairment due, in part, to a disproportionate burden of poverty, low literacy levels, substance abuse, poor mental health, barriers to health care services, and environmental exposures prevalent in predominantly minority urban communities in which they reside (8, 9). Biological factors, such as sex steroid hormones and female-specific factors (e.g., pregnancy, menopause), may also contribute to the pattern and magnitude of cognitive impairment in PWH (9). Combining samples of NVS and VS individuals introduces heterogeneity in cognitive function and findings from combined samples may not be generalizable to VS-PWH, a population that is expanding with the introduction of increasingly tolerable and available medication options.

As the pattern and predictors of cognitive function are likely not the same in (1) MWH and WWH as well as in (2) VS vs. NVS individuals (9), we examined heterogeneity in NP performance in the largest sample to date of VS-WWH and HIV-uninfected women. We accomplished this by applying novel machine learning methods to identify subgroups who demonstrated similar NP profiles. This approach may help guide our understanding of profiles that are associated with patterns of NP weakness. We also identified factors associated with each profile from a constellation of sociodemographic, behavioral, and clinical factors that have been found to be import distinguishing factors in prior studies (5–7), with the addition of female-specific factors (e.g., pregnancy, menopausal stage) that could not be examined in mixed-sex studies.

## Materials and Methods

### Participants

The Women's Interagency HIV Study (WIHS) is a multi-center, longitudinal, study of WWH and HIV-uninfected women. The first three waves of study enrollment occurred between October 1994 and November 1995, October 2001 and September 2002, and January 2011 and January 2013 from six sites (Brooklyn, Bronx, Chicago, DC, Los Angeles, and San Francisco). A more recent wave of enrollment occurred at sites in the southern US (Chapel Hill, Atlanta, Miami, Birmingham, and Jackson) between October 2013 and September 2015. Study methodology including recruitment procedures and eligibility criteria, training, and quality assurance procedures were previously published (10–12). This analysis was restricted to all participants completing the first NP test battery. NP data for the initial six sites were collected between 2009 and 2011, while NP data from the southern sites were collected between 2013 and 2015.

### Neuropsychological (NP) Test Battery and Outcomes

The NP test battery included the Hopkins Verbal Learning Test-Revised (HVLT-R; outcomes: trial 1 learning, total learning, delayed free recall, percent retention, recognition), Letter-Number Sequencing (LNS; outcomes: total correct on the working memory and attention conditions), Trail Making Test (TMT; outcomes: time to complete Parts A and B), Stroop (outcome: time to complete Trials 1 [color reading], 2 [color naming], and 3 [color-word]), Symbol Digit Modalities Test (SDMT; outcome: total correct), Letter-guided verbal fluency (Controlled Oral Word Associations Test (COWAT; outcome: total correct words generated across three trials [F, A, S]), Animal fluency (outcome: total correct animals generated), and Grooved Pegboard (GPEG; outcomes: time to completion, dominant, and non-dominant hand). Timed outcomes were log transformed to normalize distributions and reverse scored so higher equated to better performance. Demographically-adjusted T-scores were calculated for each outcome (13, 14). T-scores are normalized to have an average of 50 and a standard deviation of 10. Mean T-scores >55 were considered high performing, between 45 and 55 were considered within the normal range, <45 were considered as weaknesses, and those <40 were considered impaired.

### Factors Associated With NP Profiles

Factors of interest were based on prior NP WIHS studies (13, 14) and included: clinic site; enrollment wave; sociodemographic, mental health, behavioral, clinical, and female-specific factors; and common non-ART medications with known neurocognitive adverse effects (NCAEs) (15, 16). Sociodemographic factors included age, education, WRAT-III reading subscale score, race/ethnicity, employment status, average annual household income (≤$12,000), and health insurance status. Mental health factors included depressive symptoms (Center for Epidemiological Studies Depression scale [CES-D] ≤16]), perceived stress (perceived stress scale [PSS]-10 top tertile cutoff), and post-traumatic stress symptoms (PTSD Checklist—Civilian Scale) (17). Behavioral factors included current smoking status, recent alcohol intake, marijuana, and crack, cocaine, and heroin use. General clinical, metabolic, and cardiovascular factors included Hepatitis C antibody positive, body mass index (BMI), non-ART medication use [e.g., NCAEs, statins, NCAE medications with a higher anticholinergic burden (16)], and history of stroke, hypertension, and diabetes mellitus. Female-specific factors included ever pregnant, history of hysterectomy and/or bilateral oophorectomy, hormonal contraceptive use, hormone therapy use, and menopausal stage [defined using the Study of Women's Health Across the Nation [SWAN] criteria (18) which is also used in previous WIHS studies (19)]. HIV-related clinical factors included HIV RNA, nadir and current CD4^+^ T lymphocyte count, ART use and adherence, duration of ART use, and previous AIDS diagnosis.

### Statistical Analyses

All 17 NP measures were used to find groups of similar cognitive profiles within each participant subset (VS-WWH, HIV-uninfected) utilizing Kohonen self-organizing maps (SOM) followed by clustering with MClust. SOM is an unsupervised machine learning technique used to identify patterns in high dimensional data by producing a two-dimensional grid representation consisting of multiple nodes which have a fixed position in the SOM grid along with associated participants who are mapped to that node. The coordinates of the node represent the similarity to other nodes (i.e., nodes that are closer together in the grid have similar patterns than nodes that are further away) and one node can represent multiple participants. Following the identification of the nodes, the nodes were clustered using the MClust package. Once the clustering of the nodes was completed, cluster profiles were assigned to the participants associated to that node. Profiles where the mean T-Score on all cognitive outcomes was ≥45 were combined into an “unimpaired” profile. By using SOM and MClust in sequence, we were able to achieve fine-tuned clustering based on patterns of NP performance.

Factors associated with profile membership between each impaired profile and the unimpaired profile within each group (VS-WWH, HIV-uninfected) were explored by creating Random Forest (RF) models and then extracting variable importance. The datasets were randomly separated into training (70%) and testing (30%) sets. RF models were created on the training sets using internal validation via a 10-fold resampling method repeated five times. Prior to model creation, the Synthetic Minority Over-sampling Technique (SMOTE) was used to control for bias due to any imbalance in the number of cases. Variables were removed from the model if they had low variance or if they had >30% missing data. Any missing data in the remaining variables was imputed before model creation using RF imputations and ridge regression (α size of 0.0001 for a compromise between stability and lack of bias). For comparison to previous studies we also created RF models for each group comparing the combined unimpaired and impaired profiles. Models were also validated on the testing set to confirm that they still had predictive power balanced between classes and that success of the trained models was not due to overfitting. All variables were plotted by relative variable importance based on the training set models, and attention was given to the top 10 variables in each profile.

All analysis was done using R analysis packages. SOM was achieved using the Kohonen package in R (20) and clustering was done using the MClust package (21). MClust is an R Software package used for model-based clustering using finite normal mixture modeling that provides functions for parameter estimation via the Expectation-Maximization algorithm with an assortment of covariance structures. This program identifies the best model for 10 parameterized covariance structures and chooses the best one based on the lowest Bayesian Information Criterion (BIC). The covariance structures consist of varying distributions (spherical, diagonal, or ellipsoidal), volumes (equal or variable), shapes (equal of variable), and orientation (equal or variable, only for ellipsoidal distribution). Random Forest model creation was achieved using the Caret (22) package in R. SMOTE resampling was done using the DMwR (23) package. Imputation of missing data was done using the Multivariate Imputation by Chained Equations (24) (MICE) package in R. ROC confidence intervals were calculated using the pROC package in R with 2,000 stratified bootstrap replicates (95%CI).

## Results

### Participants

Participants included 929 VS-WWH and 717 HIV-uninfected women at their first study visit with complete NP testing (Supplementary Table 1). On average, participants were 45.1 ± 9.3 years of age with 12.7 years of education. Thirty percent were from the southern WIHS sites, 69% were non-Hispanic Black, and 15% identified as Hispanic. Only 41% were employed and 48% reported having an average annual household income <$12,000/year, while 87% were currently insured. Thirty percent had depressive symptoms while 35% were identified as having higher perceived stress levels. Nineteen percent had recently used marijuana, 7% were currently using crack, cocaine, and/or heroin, and 40% were current smokers. Ninety percent reported ever having been pregnant and 41% were post-menopausal. The average T-score for all NP tests in VS-WWH and HIV- women was in the normal range between 45 and 55 (Supplementary Table 2).

### Cognitive Profiles in VS-WWH and HIV-Uninfected Women

**Figure 1:**
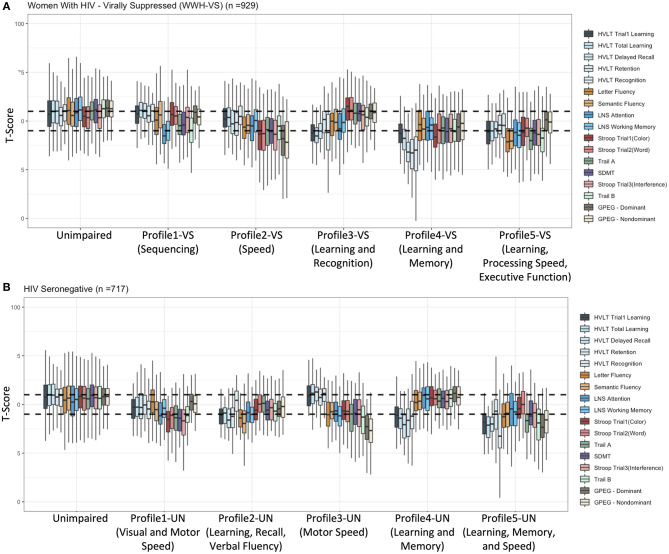
Neuropsychological profiles in **(A)** virally suppressed women with HIV(VS-WWH) and **(B)** HIV-uninfected women. HVLT-R, Hopkins Verbal Learning Test-Revised; LNS, Letter-Number Sequencing; GPEG, Grooved Pegboard.

For both VS-WWH and HIV-uninfected women, clusters of participants with similar patterns of relative performance on all 17 NP were profiled using a sequence of SOM and MClust. VS-WWH and HIV-uninfected women had good fits (entropy = 0.99) and were then assigned names based on their relative patterns of weaknesses after consultation with a clinical neuropsychologist. The profiles are visualized in Figure 1 and univariate differences between the test scores, as well as univariate differences in predictor variables, are given in Tables 1, 2 (Supplementary Tables 3, 4).

**Table 1:** Sociodemographic, clinical, and behavioral factors by subgroup of virally suppressed [VS] women with HIV [WWH].

	**Unimpaired *n* (%)**	**Profile1-VS: executive function and sequencing *n* (%)**	**Profile2-VS: processing speed, executive function, and manual speed *n* (%)**	**Profile3-VS: learning and recall *n* (%)**	**Profile4-VS: learning, memory, and speed *n* (%)**	**Profile5-VS: global weakness, processing speed *n* (%)**	***p*-value**
**Sample size**	311	129	144	137	86	122	
**Enrollment wave**							0.32
1994–1995	126 (41)	39 (30)	55 (38)	48 (35)	32 (37)	49 (40)	
2001–2002	59 (19)	29 (23)	29 (20)	17 (12)	13 (15)	26 (21)	
2011–2013	32 (10)	16 (12)	10 (7)	14 (10)	10 (12)	7 (6)	
2013–2015	94 (30)	45 (35)	50 (35)	58 (42)	31 (36)	40 (33)	
**Clinic site locations**							0.42
Chicago, DC, LA, NY, SF	212 (69)	84 (65)	95 (66)	79 (58)	54 (63)	81 (66)	
Atlanta, Birmingham, Chapel Hill, Jackson	99 (32)	45 (35)	49 (34)	58 (42)	32 (37)	41 (34)	
**Sociodemographic**							
Age, M (SD)	46.7 (8.1)	45.8 (8.6)	47.3 (8.9)	46.8 (8.5)	47.4 (8.1)	44.6 (9.3)	0.10
Years of Education, M (SD)	12.8 (3.3)	13.0 (2.9)	12.5 (3.0)	12.4 (2.7)	12.1 (2.9)	12.9 (2.9)	0.19
Race							0.05
Black non-Hispanic	202 (65)	103 (80)	104 (72)	94 (69)	65 (76)	89 (73)	
Hispanic	45 (15)	7 (5)	15 (10)	18 (13)	12 (14)	16 (13)	
Other	8 (3)	5 (4)	8 (6)	3 (2)	2 (2)	6 (5)	
White	56 (18)	14 (11)	17 (12)	22 (16)	7 (8)	11 (9)	
Annual income < $12,000 per year	126 (41)	62 (48)	80 (56)	65 (47)	60 (70)	64 (53)	<0.001
Employed	152 (49)	47 (36)	41 (29)	58 (42)	23 (27)	43 (35)	<0.001
Insured	294 (95)	125 (97)	140 (97)	135 (99)	85 (99)	115 (94)	0.16
**Mental health and substance use**							
Depressive symptoms	66 (21)	39 (30)	51 (35)	46 (34)	39 (45)	43 (35)	<0.001
Higher perceived stress	81 (26)	52 (40)	50 (35)	44 (32)	42 (49)	45 (37)	0.001
Post-traumatic stress	39 (13)	32 (25)	24 (17)	27 (20)	26 (31)	19 (16)	<0.001
Crack/Cocaine/Heroine							<0.001
Recent	16 (5)	4 (3)	9 (6)	8 (6)	6 (7)	5 (4.1)	
Former	170 (55)	58 (45)	79 (55)	66 (48)	46 (54)	35 (29)	
Never	125 (40)	67 (52)	56 (39)	63 (46)	34 (40)	82 (67)	
Marijuana use							0.002
Recent	63 (20)	23 (18)	22 (15)	25 (18)	13 (15)	9 (7)	
Former	170 (55)	63 (49)	77 (54)	68 (50)	49 (57)	54 (44)	
Never	78 (25)	43 (33)	45 (31)	44 (32)	24 (28)	59 (48)	
Smoking							0.01
Current	106 (34)	41 (32)	60 (42)	62 (45)	34 (40)	36 (30)	
Former	105 (34)	36 (28)	41 (29)	29 (21)	24 (28)	28 (23)	
Never	100 (32)	52 (40)	43 (30)	46 (34)	28 (33)	58 (48)	
Heavy drinker	20 (6)	8 (6)	8 (6)	5 (4)	5 (6)	4 (3)	0.71
**Female-specific factors**							
Ever pregnant	277 (89)	117 (91)	131 (91)	124 (91)	77 (90)	114 (93)	0.84
History of oophorectomy	54 (18)	20 (17)	20 (15)	30 (23)	25 (30)	16 (14)	0.04
Ever use oral contraceptives	260 (84)	101 (78)	119 (83)	106 (77)	70 (81)	86 (71)	0.05
Ever use hormone therapy	65 (21)	25 (19)	38 (26)	27 (29)	11 (13)	24 (20)	0.26
Menopause stage							0.19
Pre-	109 (36)	45 (36)	48 (34)	41 (30)	25 (29)	58 (48)	
Peri-	57 (19)	27 (22)	28 (20)	26 (19)	13 (15)	19 (16)	
Post-	140 (46)	52 (42)	65 (46)	68 (50)	47 (55)	45 (37)	
**HIV-related clinical characteristics**							
Nadir CD4 (cells/mL)	306 (236)	310 (237)	306 (229)	311 (218)	322 (214)	304 (206)	0.99
Current CD4 (cells/mL)	677 (343)	660 (317)	705 (312)	682 (311)	629 (287)	664 (288)	0.63
Years of effective ART	7.93 (4.75)	7.59 (4.59)	8.33 (4.29)	7.77 (4.59)	8.02 (4.49)	8.47 (4.45)	0.62
Viral Load (log)	3.45 (0.44)	3.40 (0.44)	3.41 (0.44)	3.33 (0.43)	3.35 (0.43)	3.41 (0.44)	0.14
ART adherence (≥95%)	270 (87)	119 (92)	129 (90)	120 (88)	76 (88)	109 (89)	0.70
**Non-ART use**							
NCAE	60 (19)	23 (18)	35 (24)	23 (17)	23 (27)	19 (16)	0.22
Meds with anticholinergic properties	34 (11)	14 (11)	25 (17)	16 (12)	19 (22)	11 (9.0)	0.03
Anticonvulsants	9 (3)	5 (4)	5 (4)	3 (2)	2 (2)	5 (4.1)	0.93
Statins	19 (6)	6 (5)	13 (9)	9 (7)	4 (5)	7 (5.7)	0.71
Anticholinergics	4 (1)	3 (2)	4 (3)	1 (1)	3 (4)	0 (0.0)	0.27
Antipsychotics	11 (4)	5 (4)	11 (8)	5 (4)	11 (13)	1 (0.8)	<0.001
Amphetamines	2 (1)	1 (1)	0 (0)	0 (0)	0 (0)	0 (0.0)	0.64
Opioids	24 (8)	7 (5)	5 (4)	7 (5)	5 (6)	4 (3.3)	0.39
Beta blockers	7 (2)	3 (2)	6 (4)	2 (2)	2 (2)	1 (0.8)	0.56
Gastrointestinal agents	4 (1)	2 (2)	6 (4)	2 (2)	5 (6)	2 (1.6)	0.09
Antihistamines	15 (5)	6 (5)	6 (4)	5 (4)	5 (6)	4 (3.3)	0.95
Muscle relaxants	8 (3)	1 (1)	4 (3)	2 (2)	0 (0)	1 (0.8)	0.40
Antidepressants	34 (11)	12 (9)	20 (14)	13 (10)	16 (19)	16 (13.1)	0.28

*ART, antiretrovirals; M, mean; NCAE, medications with adverse neurocognitive effects; SD, standard deviation*.

**Table 2:** Sociodemographic, clinical, and behavioral factors by subgroup of HIV-uninfected (UN) women.

	**Unimpaired *n* (%)**	**Profile1-UN: visual and motor speed *n* (%)**	**Profile2-UN: learning, recall, and verbal fluency *n* (%)**	**Profile3-UN: motor speed *n* (%)**	**Profile4-UN: learning and memory *n* (%)**	**Profile5-UN: learning, memory, and speed *n* (%)**	***p*-value**
**Sample size**	400	68	58	72	75	44	
**Enrollment wave**							0.24
1994–1995	111 (28)	19 (28)	20 (35)	25 (35)	28 (37)	17 (38.6)	
2001–2001	161 (40)	33 (49)	16 (28)	25 (35)	20 (27)	12 (27.3)	
2011–2013	44 (11)	5 (7)	5 (9)	4 (6)	7 (9)	5 (11.4)	
2013–2015	84 (21)	11 (16)	17 (29)	18 (25)	20 (27)	10 (22.7)	
**Clinic site locations**							0.40
Chicago, DC, LA, NY, SF	312 (78)	56 (82)	41 (71)	54 (75)	52 (69)	34 (77.3)	
Atlanta, Birmingham, Chapel Hill, Jackson	88 (22)	12 (18)	17 (29)	18 (25)	23 (31)	10 (23)	
**Sociodemographic**							
Age	43.3 (9.7)	42.4 (9.7)	42.6 (10.9)	43.4 (10.9)	44.5 (9.0)	44.3 (11.1)	0.78
Years of education	12.7 (3.2)	13.0 (2.9)	12.7 (2.9)	12.9 (2.6)	12.4 (2.5)	12.9 (3.7)	0.89
Race							0.45
Black non-Hispanic	266 (66)	47 (69)	40 (69.0)	48 (67)	45 (60)	33 (75)	
Hispanic	84 (21)	12 (18)	10 (17)	12 (17)	12 (16)	7 (16)	
Other	15 (4)	5 (7)	2 (3)	3 (4)	7 (9)	3 (7)	
White, non-Hispanic	35 (9)	4 (6)	6 (10)	9 (13)	11 (15)	1 (2)	
Annual income < $12,000 per year	165 (41)	31 (45.6)	27 (46.6)	38 (53)	40 (53)	25 (57)	0.13
Employed	201 (50)	22 (32.4)	24 (41.4)	22 (31)	31 (42)	17 (39)	0.006
Insured	302 (76)	55 (81)	43 (74)	61 (85)	48 (64)	35 (80)	0.07
**Mental health and substance use**							
Depressive symptoms	99 (25)	30 (44.)	19 (33)	34 (47)	17 (23)	14 (32)	<0.001
Higher perceived stress	135 (34)	29 (43)	26 (45)	34 (47)	20 (27)	15 (34)	0.05
Post-traumatic stress	77 (19)	21 (31)	12 (21)	20 (28)	13 (17)	9 (20)	0.19
**Crack/Cocaine/Heroine**							0.77
Recent	33 (8)	7 (10)	8 (14)	8 (11)	7 (9)	2 (5)	
Former	200 (50)	30 (44)	27 (47)	35 (49)	41 (55)	19 (43)	
Never	167 (42)	31 (46)	23 (40)	29 (40)	27 (36)	23 (52)	
**Marijuana use**							0.01
Recent	85 (21)	21 (31)	18 (31)	9 (13)	19 (25)	13 (30)	
Former	226 (57)	28 (41)	24 (41)	49 (68)	48 (64)	21 (48)	
Never	89 (22)	19 (28)	16 (28)	14 (19)	8 (11)	10 (23)	
**Heavy drinker**	59 (16)	10 (16)	5 (10)	9 (13)	9 (13)	3 (7)	0.57
**Smoking**							0.91
Recent	172 (43)	34 (50)	27 (47)	31 (43)	37 (49)	21 (48)	
Former	114 (29)	18 (27)	15 (26)	21 (29)	24 (32)	13 (30)	
Never	114 (29)	16 (24)	16 (28)	20 (28)	14 (19)	10 (23)	
**Female-specific factors**							
Ever pregnant	366 (92)	62 (91)	53 (91)	63 (88)	66 (88)	42 (96)	0.69
History of oopherectomy	48 (13)	7 (10)	9 (17)	6 (9)	12 (17)	5 (12)	0.68
Ever use oral contraceptives	340 (85)	51 (75)	46 (79)	61 (85)	60 (80)	35 (80)	0.34
Ever use hormone therapy	63 (16)	11 (16)	11 (19)	11 (15)	11 (15)	6 (14)	0.98
Menopause stage							0.75
Peri-	74 (19)	7 (11)	12 (22)	16 (23)	12 (16)	7 (16)	
Post-	134 (34)	20 (31)	19 (35)	25 (35)	26 (36)	18 (41)	
Pre-	182 (47)	38 (59)	24 (44)	30 (42)	35 (48)	19 (43)	
**Non-ART use**							
NCAE	53 (13)	12 (18)	8 (14)	12 (17)	8 (11)	5 (11)	0.81
Meds with anticholinergic properties	41 (10)	10 (15)	5 (9)	9 (13)	6 (8)	3 (7)	0.70
Anticonvulsants	11 (3)	0 (0)	3 (5)	3 (4)	2 (3)	0 (0)	0.40
Statins	13 (3)	4 (6)	2 (4)	2 (3)	5 (7)	2 (5)	0.70
Anticholinergics	4 (1)	2 (3)	1 (2)	2 (3)	1 (1)	0 (0)	0.65
Antipsychotics	13 (3)	4 (6)	3 (5)	1 (1)	5 (7)	1 (2)	0.47
Amphetamines	1 (0)	1 (2)	0 (0)	0 (0)	0 (0)	0 (0)	0.52
Opioids	16 (4)	4 (6)	3 (5)	6 (8)	3 (4)	2 (5)	0.72
Beta blockers	3 (1)	1 (2)	1 (2)	2 (3)	2 (3)	0 (0)	0.54
Gastrointestinal agents	5 (1)	0 (0)	1 (2)	2 (3)	1 (1)	0 (0)	0.72
Antihistamines	17 (4)	2 (3)	2 (3)	1 (1)	1 (1)	0 (0)	0.48
Muscle relaxants	7 (2)	2 (3)	1 (2)	2 (3)	1 (1)	1 (2)	0.97
Antidepressants	29 (7)	6 (9)	6 (10)	7 (10)	5 (7)	3 (7)	0.93

*ART, antiretrovirals; M, mean; NCAE, medications with adverse neurocognitive effects; SD, standard deviation*.

#### Profile Results in VS-WWH

Profiling of the 929 VS-WWH resulted in nine total clusters using an ellipsoidal multivariate mixture model with equal orientation (VVE) with an entropy of 0.99. Of these clusters, four were combined into a large “unimpaired” cluster consisting of 311 women (Figure 1A; Table 1). Of the remaining clusters:

Profiling of the 1,666 PWH resulted in three total groups from a using an ellipsoidal multivariate mixture model with equal orientation with an entropy of 0.982 (Figure 1A).

- ***Profile 1-VS*** **(*n* = 129):** ***sequencing*** indicated by weaknesses on tests of sequencing (LNS, TMT-Part B). Learning, memory, verbal fluency, processing speed, inhibition, and manual speed were preserved.
- ***Profile 2-VS*** **(*n* = 144):** ***speed*** indicated by weaknesses or impairments on most tests of speed (weak: Stroop, TMT, SDMT, GPEG-dominant hand; impaired: GPEG non-dominant hand). Verbal fluency, attention, working memory, learning, and memory were preserved.
- ***Profile 3-VS*** **(*n* = 137):** ***learning and recognition*** indicated by weak learning (HVLT-R trial 1 and total learning) and recognition (HVLT-R recognition). Retention, verbal fluency, attention, working memory, processing speed, executive functioning, and manual speed were preserved.
- ***Profile 4-VS*** **(*n* = 86):** ***learning and memory*** indicated primarily by impaired memory (HVLT-R delayed recall, recognition, retention) and weak learning (HVLT-R trial 1 and total learning), with mild weaknesses on select speeded measures (Stroop-trial 1, TMT, SDMT). Verbal fluency, attention, working memory, executive functioning, and manual speed were relatively preserved.
- ***Profile 5-VS*** **(*n* = 122):** ***learning, processing speed, attention, and executive functioning*** indicated by impaired and/or weak learning, processing speed, attention, and executive functioning (impaired: COWAT, TMT-Part A; weak: HVLT-R trial 1 and total learning, animal fluency, LNS, SDMT, Stroop-trial 3, TMT-Part B). Manual speed and memory were preserved.

#### Profile Results in HIV-Uninfected Women

Profiling of the 717 HIV-uninfected women also resulted in nine total clusters (Figure 1B; Table 2) from an ellipsoidal multivariate model with equal volume and orientation (EVE) with an entropy of 0.99. Of these clusters, four did not have mean T-scores that were <45 on any test and were therefore combined into a large “unimpaired” cluster consisting of 400 women. Of the remaining clusters:

- ***Profile 1-UN*** **(*n* = 68):** ***visual and motor speed*** indicated by weaknesses on tests of visual and motor speed (Stroop, TMT, SDMT). Learning, memory, verbal fluency, attention, working memory, and manual speed were preserved.
- ***Profile 2-UN*** **(*n* = 58):** ***learning, recall, and verbal fluency*** indicated primarily by weak learning, recall, and verbal fluency (HVLT-R trial 1, total learning, delayed recall, retention, COWAT, animal fluency). Recognition, processing speed, executive functioning, and manual speed were relatively preserved.
- ***Profile 3-UN*** **(*n* = 72):** ***manual speed*** indicated primarily by impaired manual speed (GPEG) and weak TMT. Learning and memory were spared and all other domains remained relatively preserved.
- ***Profile*** **4*****-UN*** **(*n* = 75):** ***learning and memory*** indicated by impaired recall (HVLT-R delayed recall and retention) and weak learning and recognition (HVLT-R trial 1, total learning, and recognition). All other domains were spared.
- ***Profile 5-UN*** **(*n* = 44):** ***learning, memory, speed*** indicated primarily by impaired learning and memory (HVLT-R total learning, delayed recall, and recognition) with impairments or weaknesses on select speeded tests (TMT, GPEG, Stroop-trial 3). Verbal fluency, attention, working memory, and visuo-verbal processing speed (Stroop Trials 1 and 2) were relatively preserved.

### Predictors of Cognitive Profiles

For each group of women, a RF model was created to help identify variables contributing in a non-linear fashion to distinguishing between each impaired and the unimpaired profile. An additional model was created to distinguish between all combined impairment profiles and the unimpaired profile in order to compare the differences in variables. For each model, variable importance was calculated and those that ranked as the top 10 were identified.

#### Predictors of Cognitive Profiles in VS-WWH

**Figure 2:**
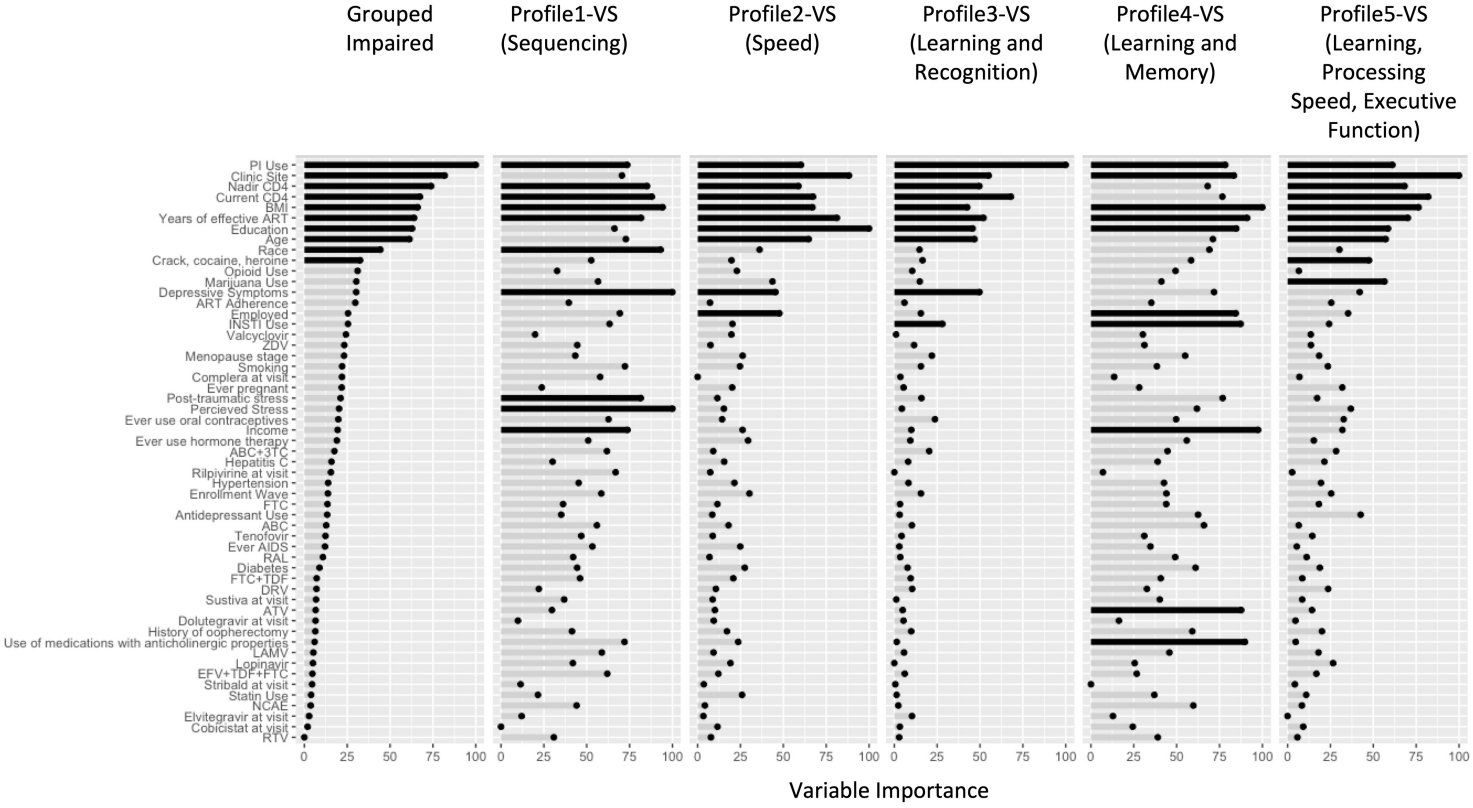
Variable Importance results from the Random Forest Models in virally suppressed women with HIV (VS-WWH). The length of the bars indicates the relative importance of the variable in that model. The top 10 variables in each model have been highlighted in black. 3TC, lamivudine; ABC, abacavir; ART, antiretroviral therapy; ATV, atazanavir; BMI, body mass index; COBI, cobicistat; DRV, darunavir; DTG, dolutegravir; EFV, efavirenz; EVG, elvitegravir; FTC, emtricitabine; INSTI, integrase inhibitor; PI, protease inhibitor; RAL, raltegravir; RPV, rilpivirine; RTV, ritonavir; TDF, tenofovir disoproxil fumarate; ZDV, Zidovudine; NCAE, non-ART medications with neurocognitive adverse effects.

In RF models (Figure 2), the top 10 variables distinguishing women in the impaired from unimpaired profiles (ROC = 0.91) included clinic site, sociodemographic factors (age, race, education), behavioral (crack, cocaine, and/or heroin use), and clinical factors (BMI, protease inhibitor [PI]-based regimen, current and nadir CD4 count, and years of cART use). Specifically, women in the impaired vs. unimpaired profiles had: a higher minority and southern site representation (non-Hispanic Black, 76 vs. 66%; southern clinics, 36 vs. 32%), less education (12.6 vs. 12.8 years), PI use(70 vs. 60%), healthy BMI of 18.5–24.9 kg/m^2^ (19 vs. 24%), and ever use of crack, cocaine, and/or heroin (49 vs. 40%). Many of these variables were also important contributors to the individual impairment profiles, although some additional variables were found to be important distinguishers. Compared to the unimpaired profile:

- ***Profile 1-VS*** **(*n* = 129):** ***sequencing*** (ROC = 0.89, Testing Accuracy = 0.89, Testing Sensitivity = 0.84, Testing Specificity = 1.0) were more likely to have a lower annual household income (48 vs. 40%), depressive symptoms (30 vs. 21%), and higher perceived (40 vs. 26%) and post-traumatic stress (25 vs. 13%).
- ***Profile 2-VS*** **(*n* = 144):** ***speed*** (ROC = 0.90, Testing Accuracy = 0.90, Testing Sensitivity = 0.85, Testing Specificity = 1.0) were more likely to be unemployed (71 vs. 51%) and have depressive symptoms (35 vs. 21%).
- ***Profile 3-VS*** **(*n* = 137):** ***learning and recognition*** (ROC = 0.90, Testing Accuracy = 0.88, Testing Sensitivity = 0.83, Testing Specificity = 1.0) were more likely to have depressive symptoms (34 vs. 21%) and be on an integrase inhibitor (INSTI)-based regimen (27 vs. 19%).
- ***Profile 4-VS*** **(*n* = 86):** ***learning and memory*** (ROC = 0.88, Testing Accuracy = 0.89, Testing Sensitivity = 0.87, Testing Specificity = 0.96) were more likely to be unemployed (73 vs. 51%, *P*<0.001), have a lower annual household income (70 vs. 40%), be on INSTI-based regimen (28 vs. 19%), atazanavir (ATZ, 24 vs. 15%), and non-ART medications with higher anticholinergic burden (22 vs. 11%).
- ***Profile 5-VS*** **(*n* = 122):** ***learning, processing speed, and executive function*** (ROC = 0.91, Testing Accuracy = 0.86, Testing Sensitivity = 0.81, Testing Specificity = 1.0) were less likely to currently use marijuana (15 vs. 20%).

#### Predictors of Cognitive Profiles in HIV-Uninfected Women

**Figure 3:**
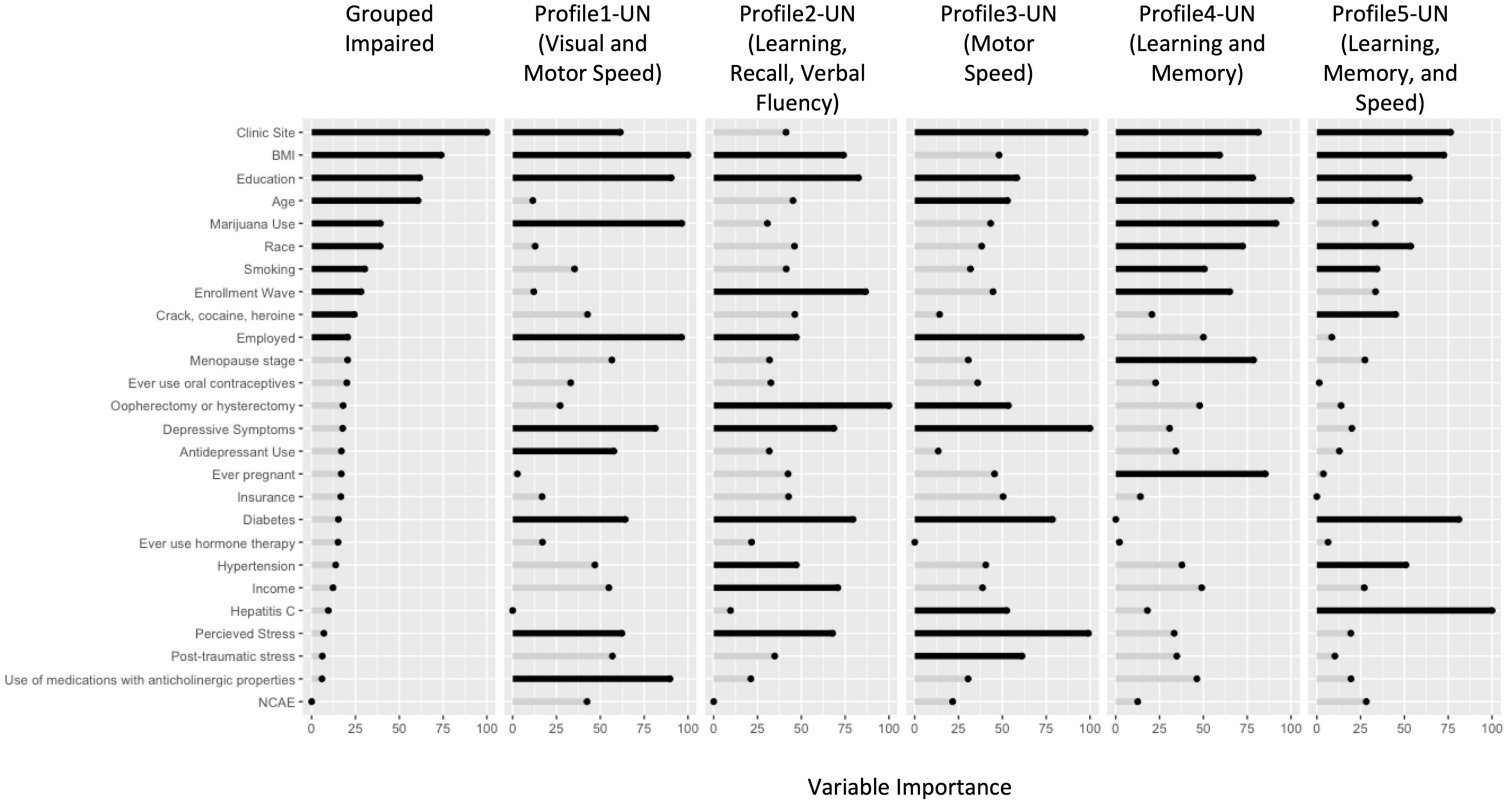
Variable Importance results from the Random Forest Models in HIV-uninfected (UN) women. The length of the bars indicates the relative importance of the variable in that model. The top 10 variables in each model have been highlighted in black. BMI, body mass index; NCAE, non-ART medications with neurocognitive adverse effects; OOPH, oopherectomy.

In RF models (Figure 3), the top 10 variables distinguishing the impaired profiles from unimpaired profiles (ROC = 0.93) included: clinic site, cohort wave, sociodemographic factors (age, education, race, employment status), behavioral (smoking, marijuana, crack, cocaine, and/or heroin use), and clinical factors (BMI). Many of these factors were also important contributors to the individual impairment profiles, with some differences. Compared to women in the unimpaired profile:

- ***Profile 1-UN*** **(*n* = 68):** ***visual and motor speed*** (ROC = 0.83, Testing Accuracy = 0.67, Testing Sensitivity = 0.90, Testing Specificity = 0.63) were more likely to have diabetes (22 vs. 16%), depressive symptoms (44 vs. 25%), high perceived stress (43 vs. 34%), use antidepressants (9 vs. 7%), and more non-ART drugs with anticholinergic properties (3 vs. 1%).
- ***Profile 2-UN*** **(*n* = 58):** ***learning, recall, and verbal fluency*** (ROC = 0.72, Testing Accuracy = 0.71, Testing Sensitivity = 1.0, Testing Specificity = 0.67) were more likely to have a lower annual household income (47 vs. 41%), have had an oophorectomy (17 vs. 13%), depressive symptoms (33 vs. 25%), high perceived stress (45 vs. 34%), diabetes (21 vs. 16%), and hypertension (36 vs. 38%).
- ***Profile 3-UN*** **(*n* = 72):** ***motor speed*** (ROC = 0.85, Testing Accuracy = 0.75, Testing Sensitivity = 1.0, Testing Specificity = 0.71) were more likely to have depressive symptoms (47 vs. 25%), perceived stress (47 vs. 34%) and post-traumatic symptoms (28 vs. 19%), HCV (18 vs. 10%), and less likely to have had an oophorectomy (9 vs. 13%).
- ***Profile*** **4*****-UN*** **(*n* = 75):** ***learning and memory*** (ROC = 0.79, Testing Accuracy = 0.76, Testing Sensitivity = 0.95, Testing Specificity = 0.73) were less likely to have ever been pregnant (88 vs. 91%).
- ***Profile 5-UN*** **(*n* = 44):** ***learning, memory, and speed*** (ROC = 0.76, Testing Accuracy = 0.73, Testing Sensitivity = 1.0, Testing Specificity = 0.70) were more likely to have diabetes (32 vs. 16%) and hypertension (53 vs. 38%).

## Discussion

We used machine learning models to identify distinct homogenous subgroups (profiles) in the largest dataset to date in VS-WWH and HIV-uninfected women. Separate patterns of cognitive performance, as well as associated factors of those patterns among each subgroup of women, were identified. The factors identified allow for screening and intervention, including potentially changing non-ART medications, as well as mental health and substance use screening and intervention.

In the context of viral suppression, we identified several profiles with distinct patterns of performance across 17 NP outcomes. While these profiles are statistically-derived, some of the profiles found here parallel commonly identified patterns in other neurological conditions or processes. Among the virally suppressed group, Profile 1-VS revealed a unique pattern reflecting exclusive weaknesses in cognitive sequencing (LNS Attention and Working Memory) and motor set-shifting (TMT-Part B). While to our knowledge, this combination of isolated deficits in cognitive sequencing and motor set-shifting has not been appreciated in other disease populations, specific deficits in cognitive sequencing/verbal working memory have been observed in individuals with schizophrenia and their first-degree relatives (25). Additionally, McDonald et al. (26) identified specific problems with motor set-shifting (TMT-Part B) in individuals with frontal lobe epilepsy. In contrast to the very specific weaknesses identified in Profile 1-VS, Profile 2-VS reflects general slowing, which is most often associated with typical (i.e., “healthy”) aging (27). Profile 3-VS, characterized by poor encoding and recognition with intact retention, is more of a typical HIV-associated profile (28) compared to mild cognitive impairment due to Alzheimer's disease (AD) (29). Profile 4-VS showed a mostly amnestic profile with some evidence of cognitive slowing, as can be observed in AD or in AD with vascular contributions (30). This profile is similar to Profile 4-UN, which reflected an amnestic profile that is often observed in typical AD (31). Profile 5-VS, showing intact memory storage and manual speed/dexterity, but weak or impaired attention, processing speed, learning, and executive functioning is similar to what is observed in individuals with diffuse frontal-subcortical small vessel disease (32). Interestingly, a profile did not emerge among VS-WWH reflecting specific motor slowing which has been linked to HIV infection. This is consistent with prior cross-sectional WIHS analyses, where motor slowing was not a prominent feature among WWH but rather verbal learning and memory (13).

Among the seronegative group, Profile1-UN was more likely to have diabetes, raising the possibility that their specific visual and motor deficits could be related to physical complications of diabetes, including diabetic retinopathy and neuropathy (33, 34). Profile2-UN reflects unique impairments on the most verbally mediated tasks (i.e., verbal learning and recall, and verbal fluencies). While we are unaware of any specific disease process or syndrome that shows the same pattern, this group of individuals has clear weakness in verbal skills, which could be due to many factors, including learning differences or damage to brain regions associated with verbally mediated tasks. Profile 3-UN, reflecting specific motor slowing, is commonly observed in individuals with basal ganglia dysfunction, such as Parkinson's disease (35). Profile 5-UN, revealing rather generalized cognitive weaknesses or impairments, but relatively preserved attention and visual processing, does not reflect any specific disease process or syndrome to our knowledge.

Even though the HIV group was virally suppressed, the dominant profiles did not fully align with the HIV-uninfected women, suggesting that HIV affects cognitive function even in the era of effective ART. There is a wealth of literature postulating neuronal damage as a result of ART agents (36), a viral reservoir that persists possibly due to poor CNS penetration of ART (37), or even legacy effects of damage occurring earlier during infection (38). Indeed, in the VS-WWH RF model where all impaired groups were grouped together, nadir CD4 was a top predictor of group membership. This also points to how existing studies that consider impairment to be a unidimensional construct may only be able to detect differences in these variables and miss those associated more strongly with some profiles than others.

Despite different cognitive profiles among VS-WWH, the most discriminative factors between each impaired profile vs. the unimpaired profile were similar and included a number of well-established sociodemographic cognitive correlates, such as years of education, age, and race/ethnicity (39). Clinic site location also emerged, a factor that we have also seen using more standard statistical approaches in the WIHS (13, 14). The factors underlying this rather robust association is unknown but may involve neighborhood factors, such as violence and food insecurity. Additionally, common behavioral correlates of cognition emerged including illicit substance use (40, 41), which in the case of marijuana was more likely to be used in the unimpaired profile compared to the impaired profile demonstrating weaknesses in learning, processing speed, and executive function (Profile 5-VS). This finding is consistent with some studies demonstrating the protective effects of marijuana use on cognition in PWH (42). We also found common clinical correlates of cognition that distinguished cognitive profiles among VS-WWH including BMI (43, 44) and PI use (45). Likely proxies of HIV disease burden, including nadir CD4 count and years of ART use, were also discriminators (38, 46, 47). In contrast, sociodemographic and medical variables were unable to distinguish cognitive profiles based on seven major cognitive domains (48).

Mental health factors also emerged as important profile discriminators among VS-WWH, including depressive and stress-related symptoms. Depressive symptoms differentiated a number of impaired profiles (4 of 5 profiles) compared to the unimpaired profile, whereas stress-related symptoms only emerged for two profiles including Profile 1-VS (sequencing) and Profile 4-VS (learning and memory). These findings align with our WIHS studies demonstrating numerous cognitive correlates of depressive symptoms (19, 49, 50), whereas stress-related symptoms related most strongly to learning and memory in the context of HIV (49, 51). Importantly, mental health factors are an unmet medical need and are modifiable targets to improve cognition in WWH (52).

INSTI use discriminated both Profile3-VS (learning and recognition) and Profile 4-VS (learning and memory) from the unimpaired profile. This finding is consistent with a number of recently published studies indicating INSTI use as a contributor to NP function. One study demonstrated an association between INSTI use and poorer learning and memory but not any other cognitive domains (53). A second study also demonstrated that switching or starting an INSTI was primarily associated with poorer learning among WWH (54). A third study demonstrated that long-term INSTI exposure distinguished two impaired profiles from an unimpaired profile (55).

Our study also allows us to investigate female-specific factors that are often ignored and identify the importance of oophorectomy and/or hysterectomy (Profile2-UN[Learning, Recall, Verbal Fluency], and Profile3-UN[Motor Speed]) and menopause status (Profile4-UN[Learning and Memory]). Interestingly, these female-specific factors only emerged as important profile discriminators among HIV-uninfected women. As the proportion of menopause-inducing and non-inducing oophorectomy and/or hysterectomy was similar across VS-WWH and HIV-uninfected women, one possible explanation is that the virus itself and clinical factors, such as ART may explain more of the variance in cognitive function in VS-WWH. However, in the absence of HIV, negative effects of oophorectomy and/or hysterectomy on cognition may become more apparent. Overall, these female-specific factors are potential contributions that are missed in other studies, which are predominately male. Future studies of women should evaluate these variables in a similar stratified form to identify potential mechanistic contributions.

The existence of distinctive patterns of cognitive performance, as well as distinct factors associated with those patterns, also adds to existing evidence of differing neuropathological mechanisms. The dominant profiles often contained patterns of weaknesses that were subclinical, yet still lower than the unimpaired profiles. In many cases, the associated factors are intervenable and should be followed up with mechanistic and longitudinal studies.

Differences between the profiles identified here and previous efforts to identify cognitive patterns can be attributed to both the methods used and the study population. To identify meaningful cognitive patterns, we used a combination of SOM and MClust, which is a slight deviation from tradition k-means clustering. The nature of k-means is that it yields clusters where the most dramatic differences are shown, which may ignore subtle differences in patterns. Even Molsberry et al. (7) and Amusan et al. (55) who used latent profile analysis using domain T-scores had their fit dominated by a high-performing and a low-performing group. Using SOM for dimension reduction on the T-scores for individual tests prevented us from following pre-conceived notions about the latent structures of cognitive domains, which have been shown to be different in HIV (56). Another reason that we may have found different profiles than prior studies is that we focused on a diverse sample of underserved lower-income, African-American and Hispanic WWH where social correlates of health are common (e.g., low educational attainment, poverty, food insecurity, etc.) (8, 57) and may lead to more heterogeneous patterns of cognitive function. Of importance, this demographic is a more accurate reflection of the HIV epidemic as opposed to the predominantly White populations evaluated in other cohorts.

The addition of machine learning models to traditional univariate statistics to identify dominating predictor variables is another distinguishing aspect of the current study. It is important to point out that RF modeling is a non-linear model, and that the variable importance measure does not take into account directionality. Therefore, it is possible to have a top predictor variable from RF that does not have *P* < 0.05 using a *t*-test. RF models are also multivariate; instead of, the predictive capabilities of variables are always observed within the context of other variables. This is important considering that none of these factors exist in isolation. This makes the model more powerful, but one limitation of this statistical approach is that it becomes more difficult to interpret and should be used as a springboard for more mechanistic studies and interventions, which is why machine learning models are often thought of as “hypothesis-generating” models.

In conclusion, in the largest sample of women to date in the United States, we have used a novel pipeline of machine learning methods to identify subgroups of patterns in NP performance and created predictive models to identify the factors that distinguish each pattern from an overall “unimpaired” group. We identified distinct patterns of cognitive weaknesses in VS-WWH that differed from the distinct patterns in HIV-uninfected women. We also identified factors that may contribute to these specific profiles as a springboard for mechanistic or interventional studies. Future studies should also investigate the stability of these profiles over time, and identify the ones, if any, that are prone to future decline.

## Data Availability Statement

Data from our study are available upon request to the MACS/WIHS Combined Cohort Study (MWCCS; https://statepi.jhsph.edu/mwccs/). This committee may be contacted via email at https://statepi.jhsph.edu/mwccs/contact-us/.

## Ethics Statement

The studies involving human participants were reviewed and approved by Institutional Review Board. The patients/participants provided their written informed consent to participate in this study.

## Author Contributions

LR has primary responsibility for final content and conceived the study idea. RD conducted the statistical analyses. RD, AB, and LR wrote the paper. All authors contributed to manuscript editing and statistical review, read, and approved the final manuscript.

## Conflict of Interest

The authors declare that the research was conducted in the absence of any commercial or financial relationships that could be construed as a potential conflict of interest.

## References

[1] Navia BA Jordan BD Price RW. The AIDS dementia complex: I. Clinical features. Ann Neurol. (1986) 19:517–24. 10.1002/ana.410190602 3729308

[2] McArthur JC Brew BJ Nath A. Neurological complications of HIV infection. Lancet Neurol. (2005) 4:543–55. 10.1016/S1474-4422(05)70165-4 16109361

[3] Vance DE Fazeli PL Dodson JE Ackerman M Talley M Appel SJ. The synergistic effects of HIV, diabetes, and aging on cognition: implications for practice and research. J Neurosci Nurs. (2014) 46:292–305. 10.1097/JNN.0000000000000074 25099061 PMC4156544

[4] Stoff DM Goodkin K Jeste D Marquine M. Redefining aging in HIV infection using phenotypes. Curr HIV/AIDS Rep. (2017) 14:184–99. 10.1007/s11904-017-0364-x 28933001 PMC5614907

[5] Lojek E Bornstein RA. The stability of neurocognitive patterns in HIV infected men: classification considerations. J Clin Exp Neuropsychol. (2005) 27:665–82. 10.1081/13803390490918426 16019643

[6] Gomez D Power C Gill MJ Koenig N Vega R Fujiwara E. Empiric neurocognitive performance profile discovery and interpretation in HIV infection. J Neurovirol. (2018) 25:72–84. 10.1007/s13365-018-0685-6 30519968

[7] Molsberry SA Cheng Y Kingsley L Jacobson L Levine AJ Martin E. Neuropsychological phenotypes among men with and without HIV disease in the multicenter AIDS cohort study. AIDS. (2018) 32:1679–88. 10.1097/QAD.0000000000001865 29762177 PMC6082155

[8] Maki PM Martin-Thormeyer E. HIV, cognition and women. Neuropsychol Rev. (2009) 19:204–14. 10.1007/s11065-009-9093-2 19430907 PMC3716452

[9] Rubin LH Neigh GN Sundermann EE Xu Y Scully EP Maki PM. Sex differences in neurocognitive function in adults with HIV: patterns, predictors, and mechanisms. Curr Psychiatry Rep. (2019) 21:94. 10.1007/s11920-019-1089-x 31522330 PMC7673651

[10] Barkan SE Melnick SL Preston-Martin S Weber K Kalish LA Miotti P. The Women's Interagency HIV Study. WIHS Collaborative Study Group. Epidemiology. (1998) 9:117–25. 10.1097/00001648-199803000-00004 9504278

[11] Bacon MC Von Wyl V Alden C Sharp G Robison E Hessol N. The Women's Interagency HIV Study: an observational cohort brings clinical sciences to the bench. Clin Diagn Lab Immunol. (2005) 12:1013–9. 10.1128/CDLI.12.9.1013-1019.2005 16148165 PMC1235804

[12] Adimora AA Ramirez C Benning L Greenblatt RM Kempf MC Tien PC. Cohort profile: the Women's Interagency HIV Study (WIHS). Int J Epidemiol. (2018) 47:393–394i. 10.1093/ije/dyy021 29688497 PMC5913596

[13] Maki PM Rubin LH Valcour V Martin E Crystal H Young M. Cognitive function in women with HIV: findings from the Women's Interagency HIV Study. Neurology. (2015) 84:231–40. 10.1212/WNL.0000000000001151 25540304 PMC4335997

[14] Rubin LH Maki PM Springer G Benning L Anastos K Gustafson D. Cognitive trajectories over 4 years among HIV-infected women with optimal viral suppression. Neurology. (2017) 89:1594–603. 10.1212/WNL.0000000000004491 28904086 PMC5634661

[15] Radtke KK Bacchetti P Anastos K Merenstein D Crystal H Karim R. Use of nonantiretroviral medications that may impact neurocognition: patterns and predictors in a large, long-term HIV cohort study. J Acquir Immune Defic Syndr. (2018) 78:202–8. 10.1097/QAI.0000000000001658 29762344 PMC5962283

[16] Rubin LH Radtke KK Eum S Tamraz B Kumanan KN Springer G. Cognitive burden of common non-antiretroviral medications in HIV-infected women. J Acquir Immune Defic Syndr. (2018) 79:83–91. 10.1097/QAI.0000000000001755 29781879 PMC6092212

[17] Rubin LH Cook JA Springer G Weber KM Cohen MH Martin EM. Perceived and post-traumatic stress are associated with decreased learning, memory, and fluency in HIV-infected women. AIDS. (2017) 31:2393–1401. 10.1097/QAD.0000000000001625 28857823 PMC5831482

[18] Greendale GA Wight RG Huang MH Avis N Gold EB Joffe H. Menopause-associated symptoms and cognitive performance: results from the study of women's health across the nation. Am J Epidemiol. (2010) 171:1214–24. 10.1093/aje/kwq067 20442205 PMC2915492

[19] Rubin LH Sundermann EE Cook JA Martin EM Golub ET Weber KM. Investigation of menopausal stage and symptoms on cognition in human immunodeficiency virus-infected women. Menopause. (2014) 21:997–1006. 10.1097/GME.0000000000000203 24496085 PMC4119867

[20] Wehrens R Buydens LMC. Self- and super-organizing maps in R: the Kohonen package. J. Stat. Softw. (2007) 21:1–19. 10.18637/jss.v021.i05

[21] Fraley C Raftery A Scrucca L. mclust: Normal Mixture Modeling for Model-Based Clustering, Classification, and Density Estimation (2014).

[22] Kuhn M. Building predictive models in R using the caret package. J. Stat. Softw. (2008) 28:1–26. 10.18637/jss.v028.i0527774042

[23] Torgo L Torgo M. Package ‘DMwR’. Comprehensive R Archive Network (2013).

[24] Van Buuren S Groothuis-Oudshoorn K. mice: Multivariate imputation by chained equations in R. J. Stat. Softw. (2011) 45:1–67. 10.18637/jss.v045.i03

[25] Horan WP Braff DL Nuechterlein KH Sugar CA Cadenhead KS Calkins ME. Verbal working memory impairments in individuals with schizophrenia and their first-degree relatives: findings from the Consortium on the Genetics of Schizophrenia. Schizophr Res. (2008) 103:218–28. 10.1016/j.schres.2008.02.014 18406578 PMC2529172

[26] McDonald CR Delis DC Norman MA Wetter SR Tecoma ES Iragui VJ. Response inhibition and set shifting in patients with frontal lobe epilepsy or temporal lobe epilepsy. Epilepsy Behav. (2005) 7:438–46. 10.1016/j.yebeh.2005.05.005 16091308

[27] Salthouse TA. The processing-speed theory of adult age differences in cognition. Psychol Rev. (1996) 103:403–28. 10.1037/0033-295X.103.3.403 8759042

[28] Scott JC Woods SP Carey CL Weber E Bondi MW Grant I. Neurocognitive consequences of HIV infection in older adults: an evaluation of the “cortical” hypothesis. AIDS Behav. (2011) 15:1187–96. 10.1007/s10461-010-9815-8 20865313 PMC3110599

[29] Milanini B Allen I Javandel S Joanna H Paul R Valcour V. Discriminant analysis of neuropsychological testing differentiates HIV-associated neurocogntive disorder from mild cognitive impairment due to Alzheimer's disease. Int. Soc. Neurovirol. (2016) 22:55.

[30] Snyder HM Corriveau RA Craft S Faber JE Greenberg SM Knopman D. Vascular contributions to cognitive impairment and dementia including Alzheimer's disease. Alzheimers Dement. (2015) 11:710–7. 10.1016/j.jalz.2014.10.008 25510382 PMC4731036

[31] Rubin LH Sundermann EE Moore DJ. The current understanding of overlap between characteristics of HIV-associated neurocognitive disorders and Alzheimer's disease. J Neurovirol. (2019) 25:661–72. 10.1007/s13365-018-0702-9 30671777 PMC6646104

[32] Wallin A Roman GC Esiri M Kettunen P Svensson J Paraskevas GP. Update on vascular cognitive impairment associated with subcortical small-vessel disease. J Alzheimers Dis. (2018) 62:1417–41. 10.3233/JAD-170803 29562536 PMC5870030

[33] Brands AM Biessels GJ De Haan EH Kappelle LJ Kessels RP. The effects of type 1 diabetes on cognitive performance: a meta-analysis. Diabetes Care. (2005) 28:726–35. 10.2337/diacare.28.3.726 15735218

[34] Allen RS Feola A Motz CT Ottensmeyer AL Chesler KC Dunn R. Retinal deficits precede cognitive and motor deficits in a rat model of type II diabetes. Invest Ophthalmol Vis Sci. (2019) 60:123–33. 10.1167/iovs.18-25110 30640976

[35] Magrinelli F Picelli A Tocco P Federico A Roncari L Smania N. Pathophysiology of motor dysfunction in Parkinson's disease as the rationale for drug treatment and rehabilitation. Parkinsons Dis. (2016) 2016:9832839. 10.1155/2016/9832839 27366343 PMC4913065

[36] Robertson K Liner J Meeker RB. Antiretroviral neurotoxicity. J Neurovirol. (2012) 18:388–99. 10.1007/s13365-012-0120-3 22811264 PMC3581315

[37] Letendre S Marquie-Beck J Capparelli E Best B Clifford D Collier AC. Validation of the CNS penetration-effectiveness rank for quantifying antiretroviral penetration into the central nervous system. Arch Neurol. (2008) 65:65–70. 10.1001/archneurol.2007.31 18195140 PMC2763187

[38] Ellis RJ Badiee J Vaida F Letendre S Heaton RK Clifford D. CD4 nadir is a predictor of HIV neurocognitive impairment in the era of combination antiretroviral therapy. AIDS. (2011) 25:1747–51. 10.1097/QAD.0b013e32834a40cd 21750419 PMC3867631

[39] Manly JJ Smith C Crystal HA Richardson J Golub ET Greenblatt R. Relationship of ethnicity, age, education, and reading level to speed and executive function among HIV+ and HIV– women: the Women's Interagency HIV Study (WIHS) neurocognitive substudy. J Clin Exp Neuropsychol. (2011) 33:853–63. 10.1080/13803395.2010.547662 21950512 PMC3383771

[40] Meyer VJ Rubin LH Martin E Weber KM Cohen MH Golub ET. HIV and recent illicit drug use interact to affect verbal memory in women. J Acquir Immune Defic Syndr. (2013) 63:67–76. 10.1097/QAI.0b013e318289565c 23392462 PMC3628722

[41] Meyer VJ Little DM Fitzgerald DA Sundermann EE Rubin LH Martin EM. Crack cocaine use impairs anterior cingulate and prefrontal cortex function in women with HIV infection. J Neurovirol. (2014) 20:352–61. 10.1007/s13365-014-0250-x 24760360 PMC4090256

[42] Saloner R Campbell LM Serrano V Montoya JL Pasipanodya E Paolillo EW. Neurocognitive superaging in older adults living with HIV: demographic, neuromedical and everyday functioning correlates. J Int Neuropsychol Soc. (2019) 25:507–19. 10.1017/S1355617719000018 30890191 PMC6705613

[43] Gustafson DR Mielke MM Tien PC Valcour V Cohen M Anastos K. Anthropometric measures and cognition in middle-aged HIV-infected and uninfected women. The Women's Interagency HIV Study. J Neurovirol. (2013) 19:574–85. 10.1007/s13365-013-0219-1 24338243 PMC3957488

[44] Rubin LH Gustafson D Hawkins KL Zhang L Jacobson LP Becker JT. Midlife adiposity predicts cognitive decline in the prospective Multicenter AIDS Cohort Study. Neurology. (2019) 93:e261–71. 10.1212/WNL.0000000000007779 31201294 PMC6656644

[45] Rubin LH Li Y Fitzgerald KC Dastgheyb R Spence AB Maki PM. Associations between antiretrovirals and cognitive function in women with HIV. J Neuroimmune Pharmacol. (2020). 10.1007/s11481-020-09910-1. [Epub ahead of print]. 32212091 PMC7511435

[46] Stankoff B Calvez V Suarez S Bossi P Rosenblum O Conquy L. Plasma and cerebrospinal fluid human immunodeficiency virus type-1 (HIV-1) RNA levels in HIV-related cognitive impairment. Eur J Neurol. (1999) 6:669–75. 10.1046/j.1468-1331.1999.660669.x 10529754

[47] Valcour V Yee P Williams AE Shiramizu B Watters M Selnes O. Lowest ever CD4 lymphocyte count (CD4 nadir) as a predictor of current cognitive and neurological status in human immunodeficiency virus type 1 infection–the Hawaii aging with HIV cohort. J Neurovirol. (2006) 12:387–91. 10.1080/13550280600915339 17065131

[48] Dawes S Suarez P Casey CY Cherner M Marcotte TD Letendre S. Variable patterns of neuropsychological performance in HIV-1 infection. J Clin Exp Neuropsychol. (2008) 30:613–26. 10.1080/13803390701565225 18608689 PMC3092709

[49] Rubin LH Pyra M Cook JA Weber KM Cohen MH Martin E. Post-traumatic stress is associated with verbal learning, memory, and psychomotor speed in HIV-infected and HIV-uninfected women. J Neurovirol. (2016) 22:159–69. 10.1007/s13365-015-0380-9 26404435 PMC4783199

[50] Rubin LH Springer G Martin EM Seaberg EC Sacktor NC Levine A. Elevated depressive symptoms are a stronger predictor of executive dysfunction in HIV-infected women than men. J Acquir Immune Defic Syndr. (2019) 81:274–83. 10.1097/QAI.0000000000002029 30893126 PMC7254882

[51] Rubin LH Cook JA Weber KM Cohen MH Martin E Valcour V. The association of perceived stress and verbal memory is greater in HIV-infected versus HIV-uninfected women. J Neurovirol. (2015) 21:422–32. 10.1007/s13365-015-0331-5 25791344 PMC4562210

[52] Pence BW O'Donnell JK Gaynes BN. Falling through the cracks: the gaps between depression prevalence, diagnosis, treatment, and response in HIV care. AIDS. (2012) 26:656–8. 10.1097/QAD.0b013e3283519aae 22398574 PMC3691843

[53] O'Halloran JA Cooley SA Strain JF Boerwinkle A Paul R Presti RM. Altered neuropsychological performance and reduced brain volumetrics in people living with HIV on integrase strand transfer inhibitors. AIDS. (2019) 33:1477–83. 10.1097/QAD.0000000000002236 31008801 PMC8194092

[54] O'Halloran JA Wang K Williams DW Dastgheyb R Fitzgerald K Kamkwalala A. Integrase inhibitor start or switch impacts learning in women with HIV. In: Conference on Retroviruses and Opportunistic Infections. Boston, MA (2020).33394812

[55] Amusan P Power C Gill MJ Gomez D Johnson E Rubin LH. Lifetime antiretroviral exposures and neurocognitive impairment in HIV. J Neurovirol. (2020) 26:743–53. 10.1007/s13365-020-00870-z 32720232

[56] May PE Heithoff AJ Wichman CS Phatak VS Moore DJ Heaton RK. Assessing cognitive functioning in people living with HIV (PLWH): factor analytic results from CHARTER and NNTC cohorts. J Acquir Immune Defic Syndr. (2020) 83:251–9. 10.1097/QAI.0000000000002252 31913991 PMC7051833

[57] Rubin LH Maki PM. Neurocognitive complications of HIV infection in women: insights from the WIHS cohort. Curr Top Behav Neurosci. (2019). 10.1007/7854_2019_101 31396894

