# Supplementary material for: Patterns and Predictors of Cognitive Function Among Virally Suppressed Women With HIV
Source: Front Neurol. 2021 Feb 11;12:604984. doi: 10.3389/fneur.2021.604984 (PMC7928382; doi:10.3389/fneur.2021.604984)
Supplement: Supplementary file 1 [file Data_Sheet_1.docx]

Supplementary Tables

# Supplemental Table 1

Sociodemographic, clinical, and behavioral factors in the total sample and by subgroup (virally suppressed [VS] women with HIV [WWH] and HIV-uninfected women).

|  | HIV-uninfected  n (%) | VS-WWH  n (%) | *P*-value |
| --- | --- | --- | --- |
| ***Sample size*** | 717 | 929 |  |
| ***Enrollment Wave*** |  |  | <0.001 |
| 1994-1995 | 220 (31) | 349 (38) |  |
| 2001-2002 | 267 (37) | 173 (19) |  |
| 2011-2013 | 70 (10) | 89 (9) |  |
| 2013-2015 | 160 (22) | 318 (34) |  |
| ***Clinic site locations*** |  |  | <0.001 |
| Chicago, DC, LA, NY, SF | 549 (77) | 605 (65) |  |
| Atlanta, Birmingham, Chapel Hill, Jackson | 168 (23) | 324 (35) |  |
| ***Sociodemographic*** |  |  |  |
| Age, M (SD) | 43.3 (9.9) | 46.5 (8.5) | <0.001 |
| Years of Education, M (SD) | 12.8 (3.0) | 12.7 (3.0) | 0.41 |
| Race |  |  | <0.001 |
| Black, non-Hispanic | 479 (67) | 657 (71) |  |
| Hispanic | 137 (19) | 113 (12) |  |
| Other | 35 (5) | 32 (3) |  |
| White, non-Hispanic | 66 (9) | 127 (14) |  |
| Annual income < $12,000 per year | 326 (45) | 457 (49) | 0.13 |
| Employed | 317 (44) | 364 (39) | 0.04 |
| Insured | 544 (76) | 894 (96) | <0.001 |
| ***Mental health and substance use*** |  |  |  |
| Depressive symptoms | 213 (30) | 284 (31) | 0.70 |
| Higher perceived stress | 259 (36) | 314 (34) | 0.33 |
| Post-traumatic stress | 152 (21) | 167 (18) | 0.10 |
| Crack/cocaine/heroin use |  |  | 0.005 |
| Recent | 65 (9) | 48 (5) |  |
| Former | 352 (49) | 454 (49) |  |
| Never | 300 (42) | 427 (46) |  |
| Marijuana use |  |  | <0.001 |
| Recent | 165 (23) | 155 (17) |  |
| Former | 396 (55) | 481 (52) |  |
| Never | 156 (22) | 293 (31) |  |
| Smoking |  |  | <0.001 |
| Recent | 322 (45) | 339 (36) |  |
| Former | 205 (29) | 263 (28) |  |
| Never | 190 (26) | 327 (35) |  |
| Heavy Drinker | 95 (14%) | 50 (6%) | <0.001 |
| ***Female-specific factors*** |  |  |  |
| Ever pregnant | 652 (91) | 840 (90) | 0.72 |
| History of oophorectomy | 87 (13) | 165 (19) | 0.001 |
| Ever use oral contraceptives | 593 (83) | 742 (80) | 0.14 |
| Ever use hormone therapy | 113 (16) | 190 (20) | 0.01 |
| Menopause stage |  |  | <0.001 |
| Pre-menopause | 328 (47) | 326 (36) |  |
| Peri-menopause | 128 (18) | 170 (19) |  |
| Post-menopause | 242 (35) | 417 (46) |  |
| ***HIV-related clinical characteristics*** |  |  |  |
| Nadir CD4 (cells/mL) | - | 308 (226) |  |
| Current CD4 (cells/mL) | - | 674 (318) |  |
| Years on ART (cells/mL) | - | 8.0 (4.6) |  |
| Detectable HIV RNA | - | 0 (0) |  |
| Viral Load (log) | - | - |  |
| ART adherence (> than 95% of the time) | - | 823 (89) |  |
| ***Non-ART use*** |  |  |  |
| NCAE | 98 (14) | 183 (20) | 0.001 |
| Meds with anticholinergic properties | 74 (1) | 119 (2) | 0.22 |
| Anticonvulsants | 19 (10) | 29(13) | 0.81 |
| Statins | 27(4) | 58 (6) | 0.04 |
| Anticholinergics | 10 (2) | 15 (2) | 0.72 |
| Antipsychotics | 27 (4) | 44 (5) | 0.56 |
| Amphetamines | 2 (<1) | 3 (<1) | 0.87 |
| Opioids | 34 (5) | 52 (6) | 0.07 |
| Beta blockers | 9 (1) | 21 (2) | 0.13 |
| Gastrointestinal agents | 9 (1) | 21 (2) | 0.17 |
| Antihistamines | 23 (3) | 41 (4) | 0.44 |
| Muscle relaxants | 14 (2) | 16 (2) | 0.94 |
| Antidepressants | 56 (8) | 111 (12) | 0.01 |

Note. ART=antiretrovirals; M=mean; NCAE=medications with adverse neurocognitive effects; SD=standard deviation

# Supplemental Table 2

Total sample and among virally suppressed (VS) women with HIV (WWH) and HIV-uninfected women.

|  | HIV-uninfected (N=717)  Mean (SD) | VS-WWH  (N=929)  Mean (SD) | *P*-value |
| --- | --- | --- | --- |
| HVLT-R Trial 1 | 50.81 (9.58) | 49.96 (9.77) | 0.07 |
| HVLT-R Total learning | 51.01 (9.46) | 50.07 (9.21) | 0.04 |
| HVLT-R Delay Free Recall | 51.02 (9.53) | 49.43 (9.28) | < 0.001 |
| HVLT-R Recognition | 50.39 (9.65) | 49.28 (11.23) | 0.03 |
| HVLT-R Percent Retention | 50.83 (9.90) | 49.27 (9.64) | 0.001 |
| Letter Fluency | 50.45 (9.79) | 49.79 (10.77) | 0.20 |
| Semantic Fluency | 50.28 (9.93) | 49.70 (10.01) | 0.24 |
| LNS Attention | 50.14 (10.08) | 48.68 (10.48) | 0.004 |
| LNS Working Memory | 50.47 (9.81) | 50.20 (10.47) | 0.59 |
| Stroop Trial1 [color] | 50.75 (9.27) | 49.38 (10.44) | 0.006 |
| Stroop Trial 2 [word] | 50.71 (9.81) | 49.71(10.06) | 0.04 |
| Trail Making Test Part A | 49.90 (9.59) | 49.14 (10.64) | 0.14 |
| Symbol Digit Modalities | 51.13 (9.87) | 50.34 (9.92) | 0.11 |
| Stroop Trial 3 [interference] | 50.25 (9.74) | 48.90 (10.94) | 0.01 |
| Trail Making Test Part B | 49.90 (9.97) | 49.233 (10.62) | 0.19 |
| Grooved Pegboard Dominant | 50.44 (9.27) | 51.20 (10.02) | 0.12 |
| Grooved Pegboard Non-Dominant | 50.37 (9.59) | 50.58 (10.20) | 0.67 |

Note. HVLT-R= Hopkins Verbal Learning Test-Revised; LNS= Letter-Number Sequencing; SD=standard deviation

# Supplemental Table 3

Profile Test Performance: virally suppressed (VS) women with HIV (WWH). *P*-value for all outcomes is <0.001

|  | **Unimpaired (N=311)** | **Profile1-VS: Executive Function and Sequencing (N=129)** | **Profile2-VS: Processing Speed, Executive Function, and Manual Speed (N=144)** | **Profile3-VS: Learning and Recall (N=137)** | **Profile4-VS: Learning, Memory, and Speed (N=86)** | **Profile5-VS: Global Weakness, Processing Speed (N=122)** |
| --- | --- | --- | --- | --- | --- | --- |
|  | Mean (SD) | Mean (SD) | Mean (SD) | Mean (SD) | Mean (SD) | Mean (SD) |
| HVLT-R Trial 1 | 54.11 (9.76) | 54.33 (7.77) | 51.86 (8.01) | 43.90 (6.49) | 43.22 (7.54) | 44.05 (9.06) |
| HVLT-R Total learning | 55.07 (7.75) | 55.31 (5.97) | 52.10 (7.60) | 42.57 (6.07) | 40.58 (7.43) | 44.53 (7.27) |
| HVLT-R Delay Free Recall | 54.56 (7.50) | 55.17 (5.64) | 49.48 (7.48) | 45.09 (5.97) | 34.19 (7.31) | 45.82 (6.08) |
| HVLT-R Recognition | 53.96 (6.51) | 53.78 (6.34) | 52.42 (6.80) | 43.63 (11.41) | 32.38 (16.35) | 47.11 (9.69) |
| HVLT-R Percent Retention | 52.51 (7.54) | 53.47 (7.21) | 48.48 (8.03) | 49.75 (7.81) | 33.29 (10.38) | 48.20 (7.86) |
| Letter Fluency | 55.81 (10.08) | 50.41 (9.88) | 46.71 (8.97) | 48.95 (8.36) | 47.67 (9.58) | 39.86 (8.88) |
| Semantic Fluency | 53.60 (9.45) | 53.43 (9.56) | 48.71 (8.84) | 49.23 (8.36) | 45.78 (9.11) | 40.24 (7.53) |
| LNS Attention | 54.43 (10.00) | 42.87 (7.85) | 47.12 (9.51) | 47.46 (9.97) | 47.51 (9.12) | 44.19 (9.85) |
| LNS Working Memory | 56.41 (9.84) | 45.00 (7.73) | 50.20 (8.45) | 49.68 (9.17) | 46.16 (9.32) | 43.33 (10.12) |
| Stroop Trial 1 [color] | 51.99 (8.59) | 53.43 (8.40) | 42.48 (10.42) | 55.66 (9.39) | 42.46 (10.20) | 44.42 (8.80) |
| Stroop Trial 2 [word] | 51.67 (8.83) | 52.81 (7.11) | 42.00 (11.18) | 56.17 (8.39) | 45.37 (10.52) | 46.36 (7.47) |
| Trail Making Test Part A | 54.81 (8.16) | 48.13 (9.31) | 44.26 (10.46) | 53.61 (8.36) | 44.48 (9.87) | 39.79 (9.55) |
| Symbol Digit Modalities | 55.54 (8.97) | 49.09 (8.27) | 44.75 (9.16) | 54.67 (7.34) | 44.93 (8.57) | 43.94 (8.56) |
| Stroop Trial 3 [interference] | 51.94 (9.77) | 51.59 (10.54) | 42.46 (10.22) | 51.91 (9.43) | 46.07 (11.04) | 44.54 (11.44) |
| Trail Making Test Part B | 56.16 (7.28) | 44.67 (10.63) | 46.51 (9.85) | 52.16 (7.54) | 44.00 (10.25) | 40.01 (9.41) |
| Grooved Pegboard Dominant | 56.12 (6.96) | 53.24 (6.33) | 40.03 (10.42) | 54.61 (6.56) | 45.97 (11.31) | 49.51 (9.30) |
| Grooved Pegboard Non-Dominant | 55.74 (6.51) | 51.97 (6.32) | 39.05 (12.32) | 54.01 (7.43) | 46.65 (10.77) | 48.48 (8.17) |
|  |  |  |  |  |  |  |
|  | n (%) | n (%) | n (%) | n (%) | n (%) | n (%) |
| **HVLT-R Trial 1** |  |  |  |  |  |  |
| T-Score > 55 | 136 (44) | 54 (42) | 43 (30) | 5 (4) | 4 (5) | 14 (12) |
| T-Score < 45 | 53 (17) | 11 (9) | 26 (18) | 77 (56) | 51 (59) | 67 (55) |
| T-Score between 45 and 55 | 122 (39) | 64 (50) | 75 (52) | 55 (41) | 31 (36) | 41 (34) |
| **HVLT-R Total learning** |  |  |  |  |  |  |
| T-Score > 55 | 154 (50) | 66 (51) | 45 (31) | 4 (3) | 2 (2) | 8 (7) |
| T-Score < 45 | 29 (9) | 6 (5) | 23 (16) | 93 (68) | 61 (71) | 62 (51) |
| T-Score between 45 and 55 | 128 (41) | 57 (44) | 76 (53) | 40 (29) | 23 (27) | 52 (43) |
| **HVLT-R Delay Free Recall** |  |  |  |  |  |  |
| T-Score > 55 | 156 (50) | 69 (54) | 39 (27) | 8 (6) | 0 (0) | 10 (8) |
| T-Score < 45 | 30 (10) | 4 (3) | 47 (33) | 69 (50) | 82 (95) | 54 (44) |
| T-Score between 45 and 55 | 125 (40) | 56 (44) | 58 (40) | 60 (44) | 4 (5) | 58 (48) |
| **HVLT-R Recognition** |  |  |  |  |  |  |
| T-Score > 55 | 162 (52) | 68 (53) | 53 (37) | 26 (19) | 2 (2) | 25 (21) |
| T-Score < 45 | 32 (10) | 12 (9) | 19 (13) | 72 (53) | 68 (79) | 45 (37) |
| T-Score between 45 and 55 | 117 (38) | 49 (38) | 72 (50) | 39 (29) | 16 (19) | 52 (43) |
| **HVLT-R Percent Retention** |  |  |  |  |  |  |
| T-Score > 55 | 116 (37) | 47 (36) | 31 (22) | 34 (25) | 2 (2) | 21 (17) |
| T-Score < 45 | 47 (15) | 10 (8) | 51 (35) | 40 (29) | 76 (88) | 44 (36) |
| T-Score between 45 and 55 | 148 (48) | 72 (56) | 62 (43) | 63 (46) | 8 (9) | 57 (47) |
| **Letter Fluency** |  |  |  |  |  |  |
| T-Score > 55 | 162 (52) | 42 (33) | 24 (17) | 28 (20) | 22 (26) | 6 (45) |
| T-Score < 45 | 47 (15) | 39 (30) | 56 (39) | 41 (30) | 36 (42) | 86 (71) |
| T-Score between 45 and 55 | 102 (33) | 48 (37) | 64 (44) | 68 (50) | 28 (33) | 30 (25) |
| **Semantic Fluency** |  |  |  |  |  |  |
| T-Score > 55 | 125 (40) | 59 (46) | 30 (21) | 40 (29) | 12 (14) | 1 (1) |
| T-Score < 45 | 54 (17) | 24 (19) | 49 (34) | 36 (26) | 37 (43) | 90 (74) |
| T-Score between 45 and 55 | 132 (42) | 46 (36) | 65 (45) | 61 (45) | 37 (43) | 31 (25) |
| **LNS Attention** |  |  |  |  |  |  |
| T-Score > 55 | 149 (48) | 8 (6) | 26 (18) | 28 (20) | 22 (26) | 14 (12) |
| T-Score < 45 | 57 (18) | 83 (64) | 65 (45) | 61 (45) | 36 (42) | 66 (54) |
| T-Score between 45 and 55 | 105 (34) | 38 (30) | 53 (37) | 48 (35) | 28 (33) | 42 (34) |
| **LNS Working Memory** |  |  |  |  |  |  |
| T-Score > 55 | 171 (55) | 14 (11) | 41 (29) | 42 (31) | 13 (15) | 15 (12) |
| T-Score < 45 | 34 (11) | 64 (50) | 39 (27) | 38 (28) | 37 (43) | 74 (61) |
| T-Score between 45 and 55 | 106 (34) | 51 (40) | 64 (44) | 57 (42) | 36 (42) | 33 (27) |
| **Stroop Trial 1[color]** |  |  |  |  |  |  |
| T-Score > 55 | 118 (38) | 51 (40) | 12 (8) | 79 (58) | 11 (13) | 15 (12) |
| T-Score < 45 | 58 (19) | 20 (16) | 81 (56) | 18 (13) | 52 (61) | 66 (54) |
| T-Score between 45 and 55 | 135 (43) | 58 (45) | 51 (35) | 40 (29) | 23 (27) | 41 (34) |
| **Stroop Trial 2 [word]** |  |  |  |  |  |  |
| T-Score > 55 | 110 (35) | 52 (40) | 14 (10) | 72 (53) | 15 (17) | 14 (12) |
| T-Score < 45 | 72 (23) | 18 (14) | 81 (56) | 13 (10) | 37 (43) | 52 (43) |
| T-Score between 45 and 55 | 129 (42) | 59 (46) | 49 (34) | 52 (38) | 34 (40) | 56 (46) |
| **Trail Making Test Part A** |  |  |  |  |  |  |
| T-Score > 55 | 156 (50) | 28 (22) | 23 (16) | 63 (46) | 10 (12) | 6 (5) |
| T-Score < 45 | 31 (10) | 45 (35) | 71 (49) | 27 (20) | 42 (49) | 87 (71) |
| T-Score between 45 and 55 | 124 (40) | 56 (43) | 50 (35) | 47 (34) | 34 (40) | 29 (24) |
| **Symbol Digit Modalities** |  |  |  |  |  |  |
| T-Score > 55 | 163 (52) | 32 (25) | 18 (13) | 68 (50) | 10 (12) | 11 (9) |
| T-Score < 45 | 37 (13) | 41 (32) | 75 (52) | 9 (7) | 44 (51) | 65 (53) |
| T-Score between 45 and 55 | 111 (36) | 56 (43) | 51 (35) | 60 (44) | 32 (37) | 46 (38) |
| **Stroop Trial 3 [interference]** |  |  |  |  |  |  |
| T-Score > 55 | 111 (36) | 41 (32) | 16 (11) | 53 (39) | 20 (23) | 16 (13) |
| T-Score < 45 | 68 (22) | 39 (30) | 83 (58) | 31 (23) | 39 (45) | 71 (58) |
| T-Score between 45 and 55 | 132 (42) | 49 (38) | 45 (31) | 53 (39) | 27 (31) | 35 (29) |
| **Trail Making Test Part B** |  |  |  |  |  |  |
| T-Score > 55 | 181 (58) | 21 (16) | 23 (16) | 44 (32) | 10 (12) | 5 (4) |
| T-Score < 45 | 17 (6) | 62 (48) | 58 (40) | 20 (15) | 45 (52) | 85 (70) |
| T-Score between 45 and 55 | 113 (36.3) | 46 (36) | 63 (44) | 73 (53) | 31 (36) | 32 (26) |
| **Grooved Pegboard Dominant** |  |  |  |  |  |  |
| T-Score > 55 | 182 (58) | 58 (45) | 10 (7) | 73 (53) | 17 (20) | 26 (21) |
| T-Score < 45 | 15 (5) | 14 (11) | 96 (67) | 8 (6) | 35 (41) | 31 (25) |
| T-Score between 45 and 55 | 114 (37) | 57 (44) | 38 (26) | 56 (41) | 34 (40) | 65 (53) |
| **Grooved Pegboard Non-Dominant** |  |  |  |  |  |  |
| T-Score > 55 | 180 (58) | 41 (32) | 10 (7) | 66 (48) | 22 (26) | 28 (23) |
| T-Score < 45 | 23 (7) | 17 (13) | 93 (65) | 17 (12) | 36 (42) | 39 (32) |
| T-Score between 45 and 55 | 108 (35) | 71 (55) | 41 (29) | 54 (39) | 28 (33) | 55 (45) |

Note. HVLT-R= Hopkins Verbal Learning Test-Revised; LNS= Letter-Number Sequencing; SD=standard deviation

# Supplemental Table 4

Profile Test Performance: HIV-uninfected (UN) women. *P*-value for all outcomes is <0.001

|  | | **Unimpaired (N=400)** | **Profile1-UN: Visual and Motor Speed (N=68)** | **Profile2-UN: Learning, Recall, and Verbal Fluency (N=58)** | **Profile3-UN: Motor Speed (N=72)** | **Profile4-UN: Learning & Memory (N=75)** | **Profile5-UN: Learning, Memory, and Speed (N=44)** |
| --- | --- | --- | --- | --- | --- | --- | --- |
|  | | Mean (SD) | Mean (SD) | Mean (SD) | Mean (SD) | Mean (SD) | Mean (SD) |
| HVLT-R Trial 1 | | 54.02 (9.02) | 48.30 (7.19) | 43.71 (6.25) | 54.61 (7.39) | 43.85 (7.70) | 40.52 (7.54) |
| HVLT-R Total learning | | 54.59 (8.29) | 48.74 (6.95) | 44.29 (5.44) | 55.43 (7.00) | 42.63 (6.26) | 37.87 (8.35) |
| HVLT-R Delay Free Recall | | 55.12 (6.95) | 49.57 (7.94) | 42.96 (6.45) | 55.63 (6.55) | 39.16 (8.23) | 39.23 (7.38) |
| HVLT-R Recognition | | 53.15 (7.08) | 46.71 (7.71) | 51.35 (7.53) | 54.75 (5.43) | 42.75 (11.46) | 35.56 (13.76) |
| HVLT-R Percent Retention | | 54.10 (7.96) | 50.58 (8.61) | 43.10 (7.20) | 53.69 (6.70) | 39.98 (10.41) | 45.57 (13.10) |
| Letter Fluency | | 52.60 (9.46) | 51.24 (10.09) | 43.20 (6.70) | 44.85 (8.61) | 52.25 (8.30) | 45.41 (10.43) |
| Semantic Fluency | | 53.38 (9.38) | 47.16 (8.16) | 40.55 (9.82) | 46.52 (7.76) | 51.72 (7.52) | 43.45 (9.48) |
| LNS Attention | | 51.83 (10.46) | 45.55 (7.58) | 46.32 (7.49) | 46.39 (7.61) | 53.98 (8.39) | 46.55 (12.44) |
| LNS Working Memory | | 53.17 (9.75) | 47.40 (6.44) | 44.55 (6.90) | 43.87 (8.92) | 53.08 (8.26) | 44.86 (9.83) |
| Stroop Trial1[color] | | 53.46 (8.70) | 41.15 (7.39) | 47.48 (7.09) | 46.14 (8.95) | 53.57 (7.19) | 47.99 (8.37) |
| Stroop Trial 2 [word] | | 52.56 (10.08) | 43.06 (7.55) | 50.34 (7.00) | 45.50 (10.01) | 53.81 (5.93) | 49.45 (9.58) |
| Trail Making Test Part A | | 52.77 (8.35) | 43.11 (8.98) | 49.91 (7.18) | 42.68 (9.99) | 52.77 (6.96) | 40.99 (10.40) |
| Symbol Digit Modalities | | 54.83 (9.13) | 41.86 (7.95) | 47.03 (8.74) | 45.25 (8.82) | 52.18 (6.12) | 45.03 (8.44) |
| Stroop Trial 3 [inteference] | | 53.32 (8.62) | 40.78 (9.11) | 48.11 (6.79) | 46.36 (11.10) | 51.51 (7.81) | 43.99 (9.38) |
| Trail Making Test Part B | | 53.12 (8.56) | 44.54 (9.16) | 46.61 (7.87) | 42.23 (10.50) | 53.66 (6.48) | 39.48 (10.89) |
| Grooved Pegboard Dominant | | 53.68 (6.22) | 51.15 (5.31) | 47.67 (6.88) | 37.20 (9.32) | 55.43 (5.85) | 36.78 (11.24) |
| Grooved Pegboard Non-Dominant | | 53.71 (6.78) | 50.08 (6.26) | 48.98 (7.27) | 36.0 (10.46) | 54.05 (6.53) | 39.58 (11.12) |
|  | |  |  |  |  |  |  |
|  | | n (%) | n (%) | n (%) | n (%) | n (%) | n (%) |
| **HVLT-R Trial 1** | | |  |  |  |  |  |
| T-Score > 55 | 176 (44) | | 10 (15) | 2 (3) | 34 (47) | 6 (8) | 1 (2) |
| T-Score < 45 | 61 (15) | | 21 (31) | 33 (57) | 3 (4) | 39 (52) | 32 (73) |
| T-Score between 45 and 55 | 163 (41) | | 37 (54) | 23 (40) | 35 (49) | 30 (40) | 11 (25) |
| **HVLT-R Total learning** | | |  |  |  |  |  |
| T-Score > 55 | 197 (49) | | 12 (18) | 0 (0) | 39 (54) | 1 (1) | 0 (0) |
| T-Score < 45 | 49 (12) | | 25 (37) | 28 (48) | 5 (7) | 45 (60) | 36 (82) |
| T-Score between 45 and 55 | 154 (39) | | 31 (46) | 30 (52) | 28 (39) | 29 (39) | 8 (18) |
| **HVLT-R Delay Free Recall** |  | |  |  |  |  |  |
| T-Score > 55 | 196 (49) | | 19 (28) | 2 (3) | 42 (58) | 1 (1) | 0 (0) |
| T-Score < 45 | 27 (7) | | 23 (34) | 39 (67) | 3 (4) | 56 (75) | 35 (80) |
| T-Score between 45 and 55 | 177 (44) | | 26 (38) | 17 (29) | 27 (38) | 18 (24) | 9 (21) |
| **HVLT-R Recognition** |  | |  |  |  |  |  |
| T-Score > 55 | 192 (48) | | 9 (13) | 22 (38) | 40 (56) | 6 (8) | 3 (7) |
| T-Score < 45 | 56 (14) | | 28 (41) | 11 (19) | 4 (6) | 39 (52) | 33 (75) |
| T-Score between 45 and 55 | 152 (38) | | 31 (46) | 25 (43) | 28 (39) | 30 (40) | 8 (18) |
| **HVLT-R Percent Retention** |  | |  |  |  |  |  |
| T-Score > 55 | 164 (41) | | 16 (24) | 2 (3) | 28 (39) | 3 (4) | 8 (18) |
| T-Score < 45 | 37 (9) | | 17 (25) | 34 (59) | 6 (8) | 49 (65) | 20 (46) |
| T-Score between 45 and 55 | 199 (50) | | 35 (52) | 22 (38) | 38 (53) | 23 (31) | 16 (36) |
| **Letter Fluency** |  | |  |  |  |  |  |
| T-Score > 55 | 150 (38) | | 25 (37) | 3 (5) | 11 (15) | 28 (37) | 7 (16) |
| T-Score < 45 | 89 (22) | | 14 (21) | 39 (67) | 37 (51) | 11 (15) | 24 (55) |
| T-Score between 45 and 55 | 161 (40) | | 29 (43) | 16 (28) | 24 (33) | 36 (48) | 13 (30) |
| **Semantic Fluency** |  | |  |  |  |  |  |
| T-Score > 55 | 164 (41) | | 13 (19) | 3 (5) | 11 (15) | 22 (29) | 5 (11) |
| T-Score < 45 | 75 (19) | | 28 (41) | 39 (67) | 27 (38) | 12 (16) | 21 (48) |
| T-Score between 45 and 55 | 161 (40) | | 27 (40) | 16 (28) | 34 (47) | 41 (55) | 18 (41) |
| **LNS Attention** | | |  |  |  |  |  |
| T-Score > 55 | 144 (36) | | 9 (13) | 8 (14) | 10 (15) | 35 (47) | 12 (27) |
| T-Score < 45 | 111 (28) | | 37 (54) | 26 (45) | 31 (43) | 11 (15) | 19 (43) |
| T-Score between 45 and 55 | 145 (36) | | 22 (32) | 24 (41) | 31 (43) | 29 (39) | 13 (30) |
| **LNS Working Memory** | | |  |  |  |  |  |
| T-Score > 55 | 158 (40) | | 8 (12) | 4 (7) | 6 (8) | 34 (45) | 8 (18) |
| T-Score < 45 | 75 (19) | | 24 (35) | 30 (52) | 41 (57) | 14 (19) | 21 (48) |
| T-Score between 45 and 55 | 167 (42) | | 36 (53) | 24 (41) | 25 (35) | 27 (36) | 15 (34) |
| **Stroop Trial 1[color]** |  | |  |  |  |  |  |
| T-Score > 55 | 174 (44) | | 0 (0) | 8 (14) | 9 (13) | 31 (41) | 10 (23) |
| T-Score < 45 | 63 (16) | | 43 (63) | 21 (36) | 27 (38) | 9 (12) | 16 (36) |
| T-Score between 45 and 55 | 163 (41) | | 25 (37 | 29 (50) | 36 (50) | 35 (47) | 18 (41) |
| **Stroop Trial 2 [word]** | | |  |  |  |  |  |
| T-Score > 55 | 169 (42) | | 2 (3) | 15 (26) | 9 (13) | 30 (40) | 13 (30) |
| T-Score < 45 | 75 (19) | | 35 (52) | 14 (24) | 31 (43) | 4 (5) | 11 (25) |
| T-Score between 45 and 55 | 156 (39) | | 31 (46) | 29 (50) | 32 (44) | 41 (55) | 20 (46) |
| **Trail Making Test Part A** |  | |  |  |  |  |  |
| T-Score > 55 | 154 (39) | | 6 (9) | 14 (24) | 5 (7) | 29 (39) | 4 (9) |
| T-Score < 45 | 65 (16) | | 39 (57) | 13 (22) | 35 (49) | 9 (12) | 33 (75) |
| T-Score between 45 and 55 | 181 (45) | | 23 (34) | 31 (53) | 32 (44) | 37 (49) | 7 (16) |
| **Symbol Digit Modalities** |  | |  |  |  |  |  |
| T-Score > 55 | 196 (49) | | 3 (4) | 7 (12) | 8 (11) | 28 (37) | 4 (9) |
| T-Score < 45 | 42 (11) | | 43 (63) | 25 (43) | 35 (49) | 12 (16) | 25 (57) |
| T-Score between 45 and 55 | 162 (41) | | 22 (32) | 26 (45) | 29 (40) | 35 (47) | 15 (34) |
| **Stroop Trial 3 [inteference]** |  | |  |  |  |  |  |
| T-Score > 55 | 172 (43) | | 3 (4) | 11 (19) | 14 (19) | 25 (33) | 4 (9) |
| T-Score < 45 | 61 (15) | | 47 (69) | 17 (29) | 30 (42) | 11 (15) | 20 (46) |
| T-Score between 45 and 55 | 167 (42) | | 18 (27) | 30 (52) | 28 (39) | 39 (52) | 20 (46) |
| **Trail Making Test Part B** |  | |  |  |  |  |  |
| T-Score > 55 | 168 (42) | | 6 (9) | 8 (14) | 5 (7) | 30 (40) | 0 (0) |
| T-Score < 45 | 67 (17) | | 36 (53) | 25 (43) | 41 (57) | 6 (8) | 28 (64) |
| T-Score between 45 and 55 | 165 (41) | | 26 (38) | 25 (43) | 26 (36) | 39 (52) | 16 (36) |
| **Grooved Pegboard Dominant** | | |  |  |  |  |  |
| T-Score > 55 | 172 (43) | | 18 (27) | 6 (10) | 0 (0) | 42 (56) | 1 (2) |
| T-Score < 45 | 29 (7) | | 10 (15) | 19 (33) | 58 (81) | 2 (3) | 33 (75) |
| T-Score between 45 and 55 | 199 (50) | | 40 (59) | 33 (57) | 14 (19) | 31 (41) | 10 (23) |
| **Grooved Pegboard Non-Dominant** | | |  |  |  |  |  |
| T-Score > 55 | 171 (43) | | 17 (25) | 10 (17) | 1 (1) | 35 (47) | 2 (5) |
| T-Score < 45 | 42 (11) | | 12 (18) | 19 (33) | 56 (78) | 5 (7) | 28 (64) |
| T-Score between 45 and 55 | 187 (47) | | 39 (57) | 29 (50) | 15 (21) | 35 (47) | 14 (32) |

Note. HVLT-R= Hopkins Verbal Learning Test-Revised; LNS= Letter-Number Sequencing; SD=standard deviation
